# Swamp-AI: a deep learning model for monitoring wetlands change across the globe

**DOI:** 10.1038/s41598-026-39257-1

**Published:** 2026-02-13

**Authors:** Charles S. Andros, Ian W. Conery, Taylor R. Alvarado, Katherine R. DeVore, Tristan D. Calaway, Andre S. Rovai, Jin Ikeda, Adam M. Collins, Yoko Masue-Slowey

**Affiliations:** 1 Environmental Laboratory, U.S. Army Engineer Research & Development Center (USACE-ERDC), Vicksburg, USA; 2 Coastal and Hydraulics Laboratory, U.S. Army Engineer Research & Development Center (USACE-ERDC), Vicksburg, USA; 3 Smithsonian Environmental Research Center, Edgewater, MD 21211 USA; 4 Center for Computation and Technology, Louisiana State University, Baton Rouge, USA

**Keywords:** Ecology, Ecology, Environmental sciences, Hydrology, Water resources

## Abstract

Cost-effective and rapid identification of changes to wetland extent is increasingly critical given their decline in coverage worldwide over the past several decades. In the case of highly remote or otherwise physically inaccessible wetlands, remote sensing often proves the only feasible method for long-term monitoring. Recently, remote sensing approaches incorporating machine learning and deep learning (DL) have gained prominence as tools for monitoring changes to wetland extent. Here, we present “Swamp-AI” a DL model trained on wetland locations from all over the world. We devised a unique annotation system leveraging multiple global datasets to create an annotated imagery database of wetlands drawn from across the globe. The scenes selected for annotation were carefully chosen to encompass a wide variety of wetland types, including coastal and inland systems. To account for seasonality, imagery acquired throughout the year was included. This annotated database was used to train 15 candidate DL models. The highest performing model, referred to as “Swamp-AI”, achieved averaged scores of 93.7% overall accuracy, 79.4% producer’s accuracy, 93.2% user’s accuracy, and 74.6% intersection over union across the test sites. These results suggest that Swamp-AI represents a promising generalizable tool for monitoring changes in the extent of wetlands globally.

**Supplementary Information:**

The online version contains supplementary material available at 10.1038/s41598-026-39257-1.

## Introduction

Wetlands constitute one of the most valuable yet ecologically vulnerable natural resources throughout the world. They provide a broad suite of ecosystem services such as water storage, nutrient and contaminant attenuation, shoreline protection, carbon sequestration as well as habitat for diverse biota at multiple trophic levels^1–3^. However, wetlands are also a fragile resource and are sensitive to both environmental factors and anthropogenic activity^4–7^. In response, wetland restoration activities have been undertaken throughout the world, and which have demonstrated wetlands’ recovery and slowed their decline in global coverage^8^. The ongoing threat to the wetlands highlights the urgency to improve wetland monitoring systems to better detect changes in wetland extent and inform ongoing and future wetlands restoration efforts^3,9,10^.

The traditional approach for mapping and monitoring wetland extent and coverage requires field sampling campaigns, which in turn provides the most reliable data for developing and ground-truthing wetlands maps and remote sensing detection models^7,11–13^. However, these sampling campaigns can be prohibitively expensive as well as time consuming given the spatial extent of some wetlands^14^. Furthermore, wetlands tend to be located in remote and/or physically inaccessible regions, which together renders recurrent, comprehensive ground surveys of wetlands impractical^2,14^.

As an alternative to field sampling, numerous studies have demonstrated substantial progress in monitoring wetland extent using remote sensing data^2,15^. Conventional approaches for mapping wetlands with remote sensing typically use methods such as visual interpretation, index-based classification and object-based image analysis^16,17^. While effective under certain conditions, these analyses require expert knowledge of the wetlands in question and can encounter challenges in accurately classifying different types of wetlands due to the difficulty of interpreting remote sensing data. Furthermore, these site-specific tuning methods tend to be limited in their ability to generalize across diverse wetland settings, given the spectral heterogeneity and complexity of wetland landscapes^15^.

More recently, developments in machine learning (ML) and deep learning (DL) have facilitated a growing body of ML/DL classifiers designed specifically for mapping and monitoring wetlands across diverse geographic contexts. For example, in Vietnam, DL models were developed to monitor the Tien Yen and Ba Lat estuaries by leveraging a combination of remote sensing and field data^13,18^. In China, DL models were utilized to monitor the Yellow River and Liaohe estuaries^19,20^. In the Czech Republic, researchers trained a DL model to identify wetlands on historical topographical maps from 1951 to 1971^21,22^. For Canada’s extensive wetland systems, several studies have integrated remote sensing and field data to construct ML/DL classifiers^7,11,12,14,23^. One unique study, targeting wetlands in Sweden, developed a DL approach that leverages knowledge distillation to accomplish model training without annotated training data^24^.

The expansion of DL/ML applications for automated wetland monitoring has further been promoted by the growing availability of open-access remote sensing data offered by cloud computing platforms like Google Earth Engine (GEE). These platforms host a wide variety of data spanning a broad range of spatial, spectral, and temporal resolutions. For optical data, common sources of low spatial resolution data include the MODIS and AVHRR sensors, while the Sentinel-2 mission, ALOS, and the Landsat series are common sources of medium-resolution data. The IKONOS, WorldView, Planet Labs, and Gaofen series are popular sources of high-resolution data. Complementary to optical data, Synthetic Aperture Radar (SAR) acquisitions—most notably from Sentinel-1, TerraSAR-X, and Gaofen-3—offer enhanced capacity for wetland characterization under conditions of cloud cover and varying illumination^15^. In addition, Light Detection and Ranging (LiDAR), Digital Elevation Models (DEMs) and land cover maps data have been incorporated in the analysis when they are available^15^. Notably, the integration of SAR from Sentinel-1 and optical data from Sentinel-2 has proven to be a highly successful source of remote sensing data for DL/ML approaches to wetlands mapping owing to the combination of medium spatial resolution and high temporal resolution^2,7,11,12,14,24^.

A common thread in many wetlands mapping efforts is the limited scale of the training and validation data used to develop mapping approaches. Many studies rely on data from individual wetlands or localized regions, while fewer studies attempt a global-scale approach. Global scale approaches face additional challenges, such as balancing temporal and spatial resolution, variability across ecosystems, and a lack of a unified wetlands classification system^15^. To address these challenges, some global-scale studies have compensated by limiting their investigation to specific classes of wetlands. For example, the Global Mangrove Watch provides a comprehensive map of mangrove extent and change throughout the world^25,26^. In another global-wetlands mapping effort, researchers used ML to target coastal wetlands, specifically tidal flats, tidal marshes, and mangroves, and tracked their global extent over the time period of 1999–2019^4^. Other global-scale studies have leveraged existing data products from around the world to create harmonized databases comprising multiple wetland classes. For example, the Global Lakes and Wetlands Database, first published in 2004 and later updated in 2024, integrates ground and satellite-based products into a single database containing 33 wetland classes^27,28^. Another example is the global 30 m wetland dataset with a fine classification system (GWL_FCS30D) maps, which used land-cover maps to train an ML classifier and generated global-scale wetland maps for the period of 2000–2020, comprising 8 wetland classes^9,10^.

While ML models can be trained directly on pixel-level labels, DL models generally rely on annotations provided at coarser scales, such as images, patches, or segmentation masks. Consequently, most existing DL models have been developed and validated at regional scales or for specific wetland types. While these models provide exceptional detail on their designated wetland, these regional-scale DL models are limited in their transferability to diverse geographic settings as these models tend to comprise highly specific classes not found across all wetlands^13,14,18,19,29^. To address these gaps, our objectives are twofold. First, we aim to develop a standardized labeling procedure and create a global-scale annotated images database appropriate for DL modeling. Second, we seek to design a DL model capable of identifying wetlands anywhere in the world without relying on region-specific training data, leveraging this annotated dataset as the foundation.

For our first objective, we release the Global Swamp Annotated Database (GSADB): a global-scale database of annotated wetlands data derived from Sentinel-2 imagery collected in the year 2019. To help guide the wetlands annotations without field investigations, we developed a novel labeling procedure that leverages multiple global-scale optical, lidar-based and pre-existing wetlands classification datasets. Finally, to simplify the annotations, only a single wetland class was used to define wetlands of all types.

For our second objective, we employ the GSADB to train a series of candidate DL models with the goal of identifying a model capable of segmenting wetlands globally. To develop our model, we examined a suite of candidate models that we adapted from DL models that have been used to classify wetlands in other parts of the world. The best performing model, which we dubbed “Swamp-AI”, achieved an average overall accuracy (OA) score of 93.7%, producer’s accuracy (PA) score of 79.4%, a user’s accuracy (UA) score of 93.2%, and an intersection over union (IoU) score of 74.6% across three wetland test locations.

While Swamp-AI is limited to a single wetlands class, it demonstrates good performance on many locations across the world. Further, Swamp-AI expands former probabilistic mapping of wetlands approaches beyond tidal brackish and saline marshes and mangroves^4^. Therefore, we recommend Swamp-AI as a first step for monitoring diverse wetland areas by providing general guidance as to where more detailed investigation and ground validation should be prioritized.

## Materials and methods

### Experimental design

The goal of this work was to develop a broadly generalizable DL model for wetland segmentation capable of generating reliable results across the world. The first step was to create a dataset of annotated images for developing this model. The GSADB comprised 102 annotated Sentinel-2 images taken from 34 locations and was designed to serve as the training/validation dataset. The labeling procedure leveraged the GWL_FCS30D wetlands landcover maps, a 12-meter resolution TerraSAR-X DEM, and a normalized difference vegetation index (NDVI) spectral index derived from the Sentinel-2 imagery.

To train the candidate models, the GSADB images were split into a training and validation dataset using a 90/10 split. To prevent spatial leakage between the training and validation sets, the images were split using their geographic location rather using than random sampling. This ensured that no validation image was geographically adjacent to any training image. The locations for the validation set were selected both to ensure an even 90/10 split of the data and to ensure scene diversity in the validation set. For example, one highly urbanized location was specifically selected for the validation set to ensure the model would perform well on urban scenes. The other two locations selected for the validation set represented inland and coastal wetlands, respectively. An independent test dataset was created separately from the GSADB by annotating 3-meter resolution imagery of three wildlife wetland preserves. The annotations were created using the same methodology used to create the GSADB.

**Fig. 1:**
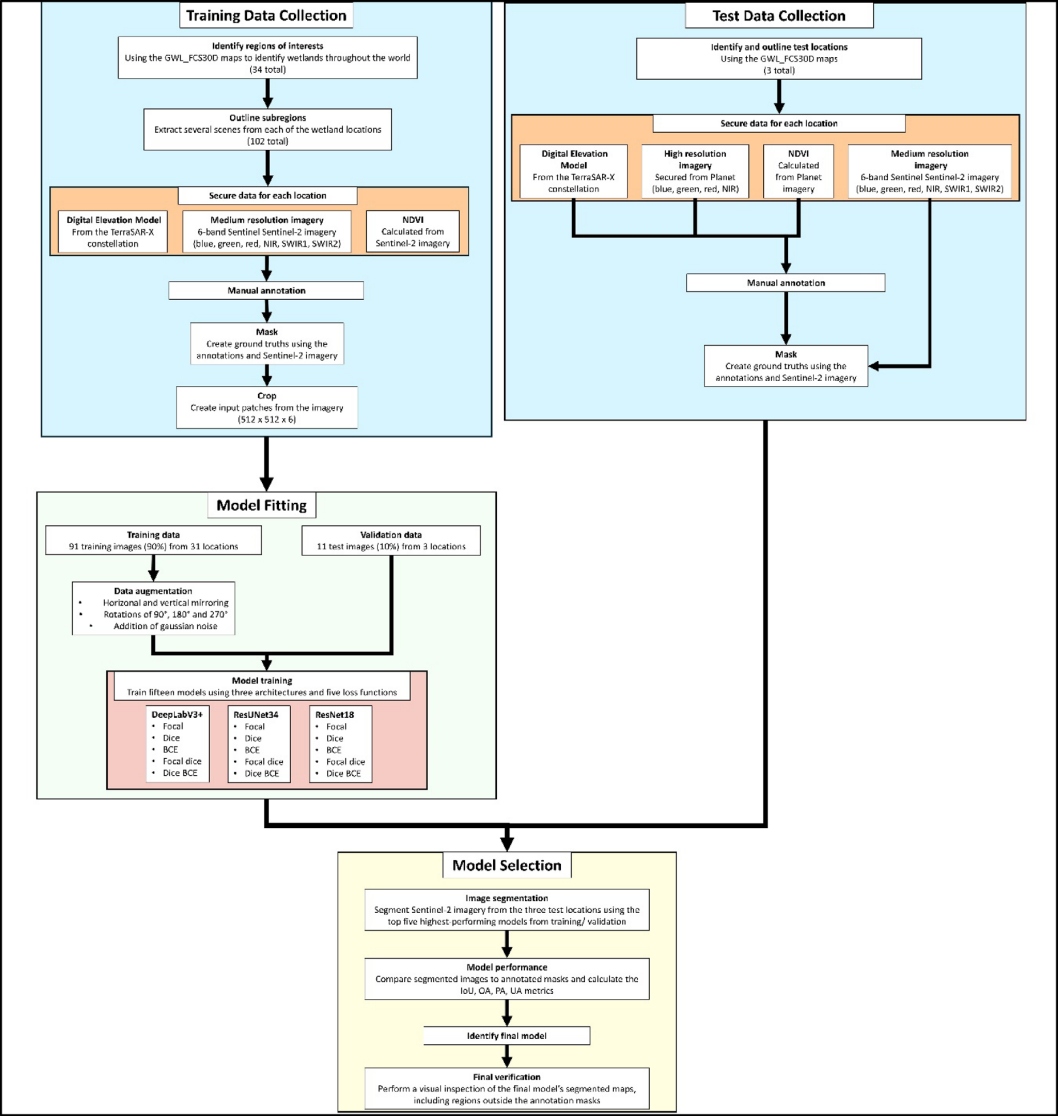
Workflow showing the curation of the GSADB, curation of the test dataset, model training and validation, and the final model selection.

A total of 15 candidate models were trained using the images in the GSADB, with the candidate models comprising a variety of DL architectures and loss functions. After model training, five of these models were selected for testing using the test dataset. For the model that achieved the highest accuracy metrics on the test set, a qualitative examination was conducted as a final check of this model’s performance. This was done by visually inspecting a segmented map generated by the final model, covering a large area beyond the annotation boundaries used for model testing. The workflow for this entire process is shown in Fig. 1.

### Curating the GSADB

To understand the annotation process used to curate the GSADB, it is necessary to define wetlands within this study. For example, the United States Army Corp of Engineers (USACE) has defined wetlands as areas that are “inundated or saturated by surface water or groundwater at a frequency and duration sufficient to support, and that under normal circumstances do support, a prevalence of vegetation typically adapted for life in saturated soil conditions”^30^. Thus, wetlands are identified based on three diagnostic criteria: hydrology, hydric soils, and hydrophytic vegetation. In this study, wetlands are simply defined as non-inundated areas that are identified by the GWL_FCS30D landcover maps as wetlands. A positive (> 0.1) NDVI value was used to help judge whether an area should be considered inundated or not. In addition, expert judgement was used to consider the Sentinel-2 imagery, as well as the terrain provided by the DEM, to make the final determinations of which pixels to label as wetlands.

**Fig. 2:**
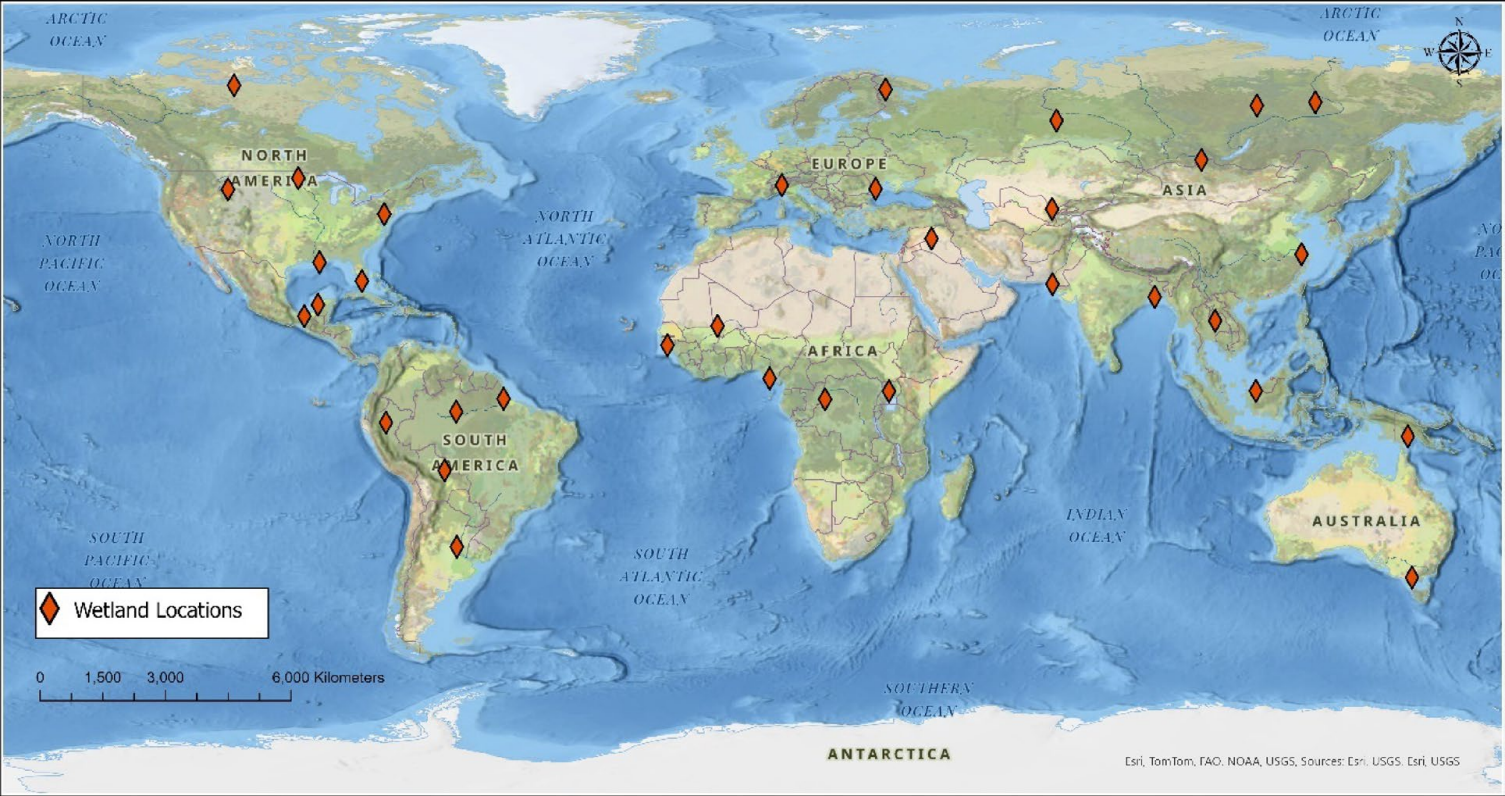
Locations selected for the GSADB. Map created in ArcGIS Pro (version 3.6.0, Esri; https://www.esri.com/) using the National Geographic Style Map basemap.

To curate the GSADB, Sentinel-2 imagery of wetlands from 2019 was collected from 34 different locations worldwide (Fig. 2). The images were exclusively drawn from the year 2019 for the sake of consistency among all images in the dataset. From the selected locations, multiple subregions were sampled and individually annotated, resulting in a total of 102 annotations. The selection process emphasized diversity in wetland environments to capture a variety of both inland and coastal regions, resulting in a total of 21 inland and 13 coastal locations. To account for seasonal variability, including ice cover and plant growth cycle, images were also selected throughout the year of 2019, with 24 images taken between January – April, 44 images taken between May – August, and 32 images taken between September – December. In addition, to improve the capacity of the classifier model to generalize to areas that are not predominantly wetlands, several locations were deliberately selected for their surface water features despite their limited wetland coverage.

All imagery was obtained from the Sentinel-2 MultiSpectral Instrument, specifically the Level-2 A surface reflectance product^31^. The Sentinel-2 sensor has 13 spectral bands, 10-meter resolution, a 5 day revisit interval, and global coverage since 2015. Most imagery was accessed through the GEE^32^ API due to its availability. Distinguishing between wetlands and open water bodies is a significant challenge in remote sensing classification^14,24^. Consequently, we adopted the band selection chosen by Luo et al. (2021) who successfully classified surface water across diverse global scenes using the red, green, blue, NIR, SWIR1 and SWIR2 Sentinel-2 bands (labeled B2, B3, B4, B8, B11, B12, respectively).

The 34 locations for annotation were identified using the GWL_FCS30D wetlands maps, which presents a global landcover map at 30-meter resolution. Represented in the GWL_FCS30D maps are 8 different wetlands categories. The maps were originally developed from 2020 imagery, then refined through time series analysis to only include wetlands that remained temporally stable between 2000 and 2022. The 2019 map was selected for wetlands location selection to ensure consistency with the Sentinel-2 imagery used for the GSADB.

To help the model generalize across both wetland and non-wetland landscapes, we also incorporated locations from the Earth Surface Water Knowledge Base^33^ to provide both globally and seasonally diverse scenes that contained water-land boundaries that are not dominated by wetlands. A total of 42 scenes were taken from 11 well-known wetland locations (i.e., Ganges River delta, Amazon River delta, etc.) while 60 scenes were taken from 23 locations not dominated by wetlands.

For each Sentinel-2 image, NDVI maps were generated to help the labelers account for seasonality and water level. The NDVI index helped distinguish fully inundated wetlands and to identify green and growing wetlands, which is important for tracking seasonality^34^. The NDVI was calculated using the following equation,

1 $$\:NDVI\:=\:\frac{{\rho\:}_{NIR}\:\:-\:\:{\rho\:}_{Red}}{{\rho\:}_{NIR}\:+\:{\rho\:}_{Red}}$$

**Fig. 3:**
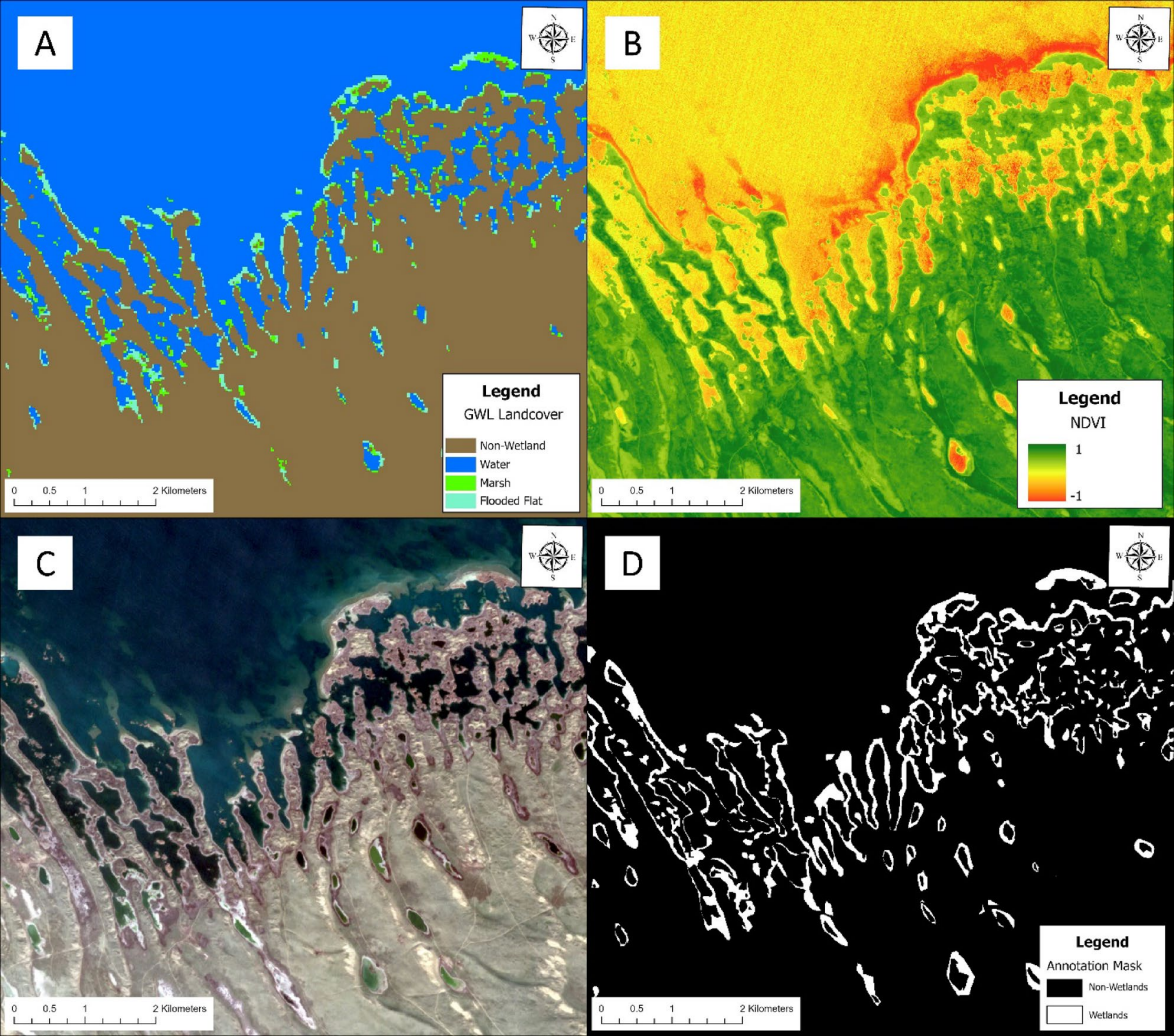
Illustrative example of the workflow used to create the annotations used in this work. Shown here is Aydar Lake, Uzbekistan overlaid with the 2019 GWL_FCS30D landcover map (**A**), a Sentinel-2 derived NDVI (**B**), Sentinel-2 imagery (**C**), and the final mask for one of the images in the GSADB (**D**). Satellite imagery was obtained from Google Earth Engine using the Harmonized Sentinel-2 MSI Level-2 A Surface Reflectance dataset (COPERNICUS/S2_SR_HARMONIZED), and processed within ArcGIS Pro (version 3.6.0, Esri; https://www.esri.com/).

Where $\:{\rho\:}_{NIR}$ is the near-infrared band and $\:{\rho\:}_{Red}$ is the visible red band^35^. Maps were carefully labeled to ensure that only active, non-inundated wetlands were labeled, typically using positive NDVI values as a screening tool. The GWL_FCS30D maps were also used during labeling to help identify the probable extent of the wetlands. Finally, a 2020 TerraSAR-X DEM at 12-meter resolution was used to provide the labelers with additional context on the terrain elevation. Together, the GWL_FCS30D, TerraSAR-X DEM and NDVI images provided the labelers with substantial context for interpreting the Sentinel-2 imagery without the assistance of a field investigation (Fig. 3).

The labeling approach employed a flexible workflow that depended on the location. When labeling an image of a wetland, the GWL_FCS30D maps were primarily used to confirm the scene as a wetland and NDVI imagery was used as a supplementary screening tool to distinguish vegetated surfaces from obvious non-wetland features such as clouds, open water, sand, or barren areas. In regions not dominated by wetlands, the GWL_FCS30D maps were used more extensively to identify candidate wetland pixels within each scene. These candidate pixels were then evaluated for consistency with expected spectral characteristics, including positive NDVI values. Pixels meeting both criteria were considered to have strong support for wetland labeling. In cases where classification remained ambiguous, topographic context from the TerraSAR-X DEM was consulted to provide additional supporting evidence and improve label confidence.

Four annotators contributed to the labeling described in this work. To ensure consistency, annotators applied a general threshold of NDVI > 0.1 to screen for vegetated wetlands, and global datasets such as the GWL_FCS30D maps and TerraSAR-X DEM were used to provide consistent spatial context across scenes. All annotations were exchanged and reviewed by at least one other annotator. Disagreements between two annotators were resolved through discussion among all four annotators to achieve consensus. Scenes for which consensus could not be reached were discarded. This workflow ensured that labeling criteria were applied consistently and reproducibly across annotators and geographic regions.

This labeling approach relies heavily on the GWL_FCS30D maps, particularly in regions where wetlands are sparse or difficult to delineate. This dependence reflects a broader challenge in global wetland mapping, where annotated reference images are limited. We selected this approach because it best supports the objective of training a deep learning model capable of identifying wetlands at the global scale, which requires geographically diverse and internally consistent annotations. Although land-cover labeling is inherently subjective, using a globally validated wetland product provides a common reference framework for both wetland and non-wetland classes. The GWL_FCS30 maps report an overall classification accuracy of 86.4%, based on 25,708 globally distributed validation samples, each confirmed through unanimous agreement among five wetland experts^9^. These maps were further refined to retain only temporally stable wetland areas, producing the GWL_FCS30D dataset^10^. Given the documented global accuracy, expert-validated reference design, and temporal-stability refinement of GWL_FCS30D, we considered it appropriate to use for guiding large-scale annotation. However, we emphasize that GSADB has inevitably inherited some of the assumptions and limitations of the GWL_FCS30D maps.

Once labeling was completed for images in the GSADB, the distribution of wetland and non-wetland classes was examined. Class proportions were calculated for each annotated image by computing the percentage of pixels belonging to the wetland class. Across the 102 images, wetlands were generally the minority class, with wetland coverage ranging from 0% to 88.8%. The average wetland proportion was 30.5% (standard deviation = 29.5%), with a median of 22.7%, indicating a substantial class imbalance with the majority class being the non-wetland class.

### Model architectures

For the candidate models, three model architectures that have been successfully utilized in other wetlands mapping studies were selected: the ResNet18, ResUNet34, and DeepLabV3 + models^18,19,29^. The ResNet model architecture was developed as a DL architecture that could go deeper (i.e., stack more layers) without suffering from vanishing / exploding gradients. The model employs stacked layers of convolutions with an added shortcut connection. The shortcut connection adds together the input to the convolution layers to the output of those same layers. This combination of stacked layers with a shortcut connection are known as a residual block. By stacking these residual blocks, models can be made very deep by avoiding the loss of too much information during the encoding process^36^. However, researchers have also shown that making models too deep can cause overfitting^37^. The ResNet18 model has been successfully employed in semantic segmentation tasks aimed at mapping coastal wetlands, making it a good candidate model for this study^19^.

The ResUNet model architecture is a merger of the ResNet and the U-Net models. The U-Net architecture was originally introduced as a segmentation model capable of handling smaller training datasets without significant losses to performance^21^. The model is characterized by a symmetrical encoder-decoder path, where feature maps from the encoder are concatenated with upsampled feature maps from the decoder. To create the ResUNet model, residual blocks are placed in the encoder of a U-Net model architecture while the U-Net decoder remains unchanged. This allows the model to go deeper using the skip connection of the residual blocks and take advantage of the high-performance of U-Net. Given the relatively small size of the GSADB training dataset, the use of the U-Net architecture for this study was also desirable given U-Net’s success in handling smaller training datasets. Finally, like the ResNet18 architecture, the ResUNet34 model demonstrates a good balance of model depth and has successfully been employed on wetlands segmentation tasks^18^.

The DeepLabV3 + model architecture is the final model examined in this study. The DeepLab series of models employ an “atrous” (with holes) convolution to reduce the loss of information caused by repeated maxpooling and downsampling operations^38^. Later iterations of DeepLab introduced the atrous spatial pyramid pooling (ASPP) module to capture multiscale contextual information^39,40^. In addition, the ASPP module allows the convolution operation to increase the field of convolution without increasing the model parameters, greatly enhancing the model’s efficiency. The DeepLabV3 + adds a decoder module to improve model predictions along object boundaries^41^. The DeepLabV3 + architecture has also been employed in wetland segmentation studies as well as studies with limited training data, making it a good candidate model for this study^33^.

### Loss functions

In additional to model architecture, the selection of loss function is an important consideration for the final model’s performance. The traditional loss function for classification tasks is the cross-entropy loss function. However, additional loss functions have been introduced to help DL models handle class imbalance in their training data, such as the focal and dice loss functions. As noted previously, class imbalance was certainly a consideration for this work as the coverage area of wetlands in the GSADB annotations ranges from 0% to 88.8% wetland, depending on the image. Therefore, a variety of loss functions were employed to address how best to utilize the GSADB for semantic segmentation.

For each of the three models we ran five separate tests, each employing a different loss function, resulting in 15 candidate models. These loss functions included, binary cross entropy (BCE), dice, focal, BCE merged with dice, and focal merged with dice.

The BCE loss function emphasizes pixel-wise accuracy and thus maximizes overall accuracy. It is defined as,

2 $$\:{\mathcal{L}}_{BCE}=\:\left\{\begin{array}{c}-log\left(\widehat{y}\right),\:\:\:\:\:\:\:\:\:\:\:\:\:\:\:\:\:\:if\:y=1\\\:-log(1-\:\widehat{y}),\:\:\:\:\:\:\:\:\:if\:y=0\end{array}\right.$$

Or alternatively,

3 $$\:{\mathcal{L}}_{BCE}=\:-\:\left[y\mathrm{log}\left(\widehat{y}\right)+\left(1-y\right)\mathrm{log}\left(1-\widehat{y}\right)\right]$$

Where $\:y$ represents the true value and $\:\widehat{y}$ the predicted value^42^. If class imbalance exists in the data, the majority class will control the losses during training and cause the model to optimize to that class. In contrast, dice loss is better suited to cases where class imbalance exists. Here we use the soft dice loss function, which is defined as,

4 $$\:{\mathcal{L}}_{dice}=1-\:\frac{2\mathrm{*}\sum\:\left(y\mathrm{*}\widehat{y}\right)+\:\in\:}{\left(\sum\:y+\sum\:\widehat{y}\right)+\:\in\:}$$

Where $\:y$ represents the true value, $\:\widehat{y}$ the predicted value, and $\:\in\:$ is a small constant that serves to prevent numerical instability in the case where $\:y+\widehat{y}=0$^43^. The dice loss measures how much overlaps exists between the predicted and the true mask and ranges from 0 to 1, where $\:{\mathcal{L}}_{dice}=0$ indicates perfect overlap and $\:{\mathcal{L}}_{dice}=1$ indicates no overlap^24,44,45^.

The focal loss function, like dice loss, is highly suited to dealing with imbalanced datasets. Lin et al. (2017) introduces two focal loss functions: a standard form and an α-balanced variant. The $\:\alpha\:$ -balanced variant was reported to yield improved accuracy over the non- $\:\alpha\:$ -balanced variant; therefore, the $\:\alpha\:$ -balanced variant was used in this work and is defined as,

5 $$\:{p}_{t}=\left\{\begin{array}{c}\widehat{y},\:\:\:\:\:\:\:\:\:\:\:\:\:\:\:\:\:\:\:\:if\:y=1\\\:1-\:\widehat{y},\:\:\:\:\:\:\:\:\:\:\:if\:y=0\end{array}\right.$$

6 $$\:{\alpha\:}_{t}=\left\{\begin{array}{c}\alpha\:,\:\:\:\:\:\:\:\:\:\:\:\:\:\:\:\:\:\:\:\:if\:y=1\\\:1-\:\alpha\:,\:\:\:\:\:\:\:\:\:\:\:if\:y=0\end{array}\right.\:$$

7 $$\:{\mathcal{L}}_{focal}=\:-{\alpha\:}_{t}\mathrm{*}{\left(1-{p}_{t}\right)}^{\gamma\:}\mathrm{*}\mathrm{l}\mathrm{o}\mathrm{g}\left({p}_{t}\right)$$

Where $\:\alpha\:$ represents a weighting factor between 0 and 1 and $\:\gamma\:$ represents a tunable focusing parameter^46^. The α and γ parameters were set to values of 0.25 and 2, respectively. These values were used to tune the focal loss function so that it would emphasize correctly classifying negative samples (i.e., non-wetlands) while reducing the influence of positive examples (i.e., wetlands). As noted previously, the wetland class constitutes the minority class (average proportion of 30.5% and a standard deviation of 29.5%). However, early models tended to overpredict the wetlands class by misclassifying non-wetland vegetation as wetlands, likely due to the strong influence of NDVI on labeling wetlands during the annotation process. While the focal loss function is expected to emphasize the minority class, which in this case represents the wetland class, the systematic overprediction of wetlands in early model runs prompted the use of a lower α value to penalize false positives in the non-wetland class more strongly. Therefore, the focal loss hyperparameters were chosen to help mitigate overprediction of wetlands and to improve discrimination between wetland and non-wetland vegetation.

The merged loss functions included the BCE merged with dice and focal merged with dice. Merging loss functions together can leverage the strengths of both loss functions and can provide a superior optimization^47^. Following the approach of Dang et al. (2020), merged losses were calculated at each step of training using both loss functions and these losses were merged into one value. For example, the merged BCE and dice loss function was defined as,

8 $$\:{\mathcal{L}}_{BC{E}_{dice}}=\:{\mathcal{L}}_{dice}+\:{\mathcal{L}}_{BCE}$$

For the merged loss functions, both terms were weighted equally, and no scaling was performed.

### Model training

Out of the 34 locations in the GSADB, 3 were selected for validation and 31 were used for model training. The train/validation split was performed using geographical location rather than random sampling to prevent spatial leakage between the training and validation sets. The locations were chosen to provide both scene diversity and to provide a 90/10 split of the 102 total images. The three locations selected for the validation set represented an urban scene, an inland wetland scene and a coastal wetland scene. Ultimately, the validation set comprised 11 images and the training set comprised 91 images. The patch size for these images was 512 × 512 pixels. The small size of the validation set was justified both to give the models as much training data as possible and because the model generalization error would be further estimated by a separate test exercise. Prior to segmentation, all pixel’s values across all bands were scaled down by a factor of 1 × 10^4^ and the pixels within each of the six bands were normalized to the range [0, 1]. The training images were also randomly selected for additional augmentation, including horizonal and vertical mirroring, rotations of 90°, 180° and 270° and adding gaussian noise to the images. A total of 15 candidate models were trained for this analysis. These models were designed using the three DL model architectures, ResUNet34, DeepLabV3+, and ResNet18, combined with each of the five loss functions, BCE, dice, focal, BCE merged with dice, and focal merged with dice. Training for all fifteen model runs was performed using the Adam optimizer^48^ with a batch size of 8, except for the DeepLabV3 + model runs which was reduced to a batch size of 4. In both cases, the batch size was as large as possible while staying within GPU memory limitations. The learning rate policy employed by Chen et al. (2017) was employed for all model runs in this study,

9 $$\:lr={lr}_{initial}\mathrm{*}{\left(1-\:\frac{epoch}{max\_epochs}\right)}^{power}$$

where *lr* is the learning rate, power was set to 0.9. For the initial learning rate ($\:{lr}_{initial}$), we conducted experiments using values of 0.001, 0.002, and 0.005. We ultimately selected 0.002 for the initial learning since it provided stable gradients during training. The total number of epochs was initially set to 300 epochs; however, all models tested this way converged by ~ 100 epochs. Therefore, to ensure consistency across model runs and to reduce the risk of overfitting, all model training runs were fixed to 100 epochs. The workflow for curating the dataset and all modeling performed is shown in Fig. 1. All model runs were performed on a machine with an NVIDIA Quadro RTX 4000 GPU.

### Model testing

**Fig. 4:**
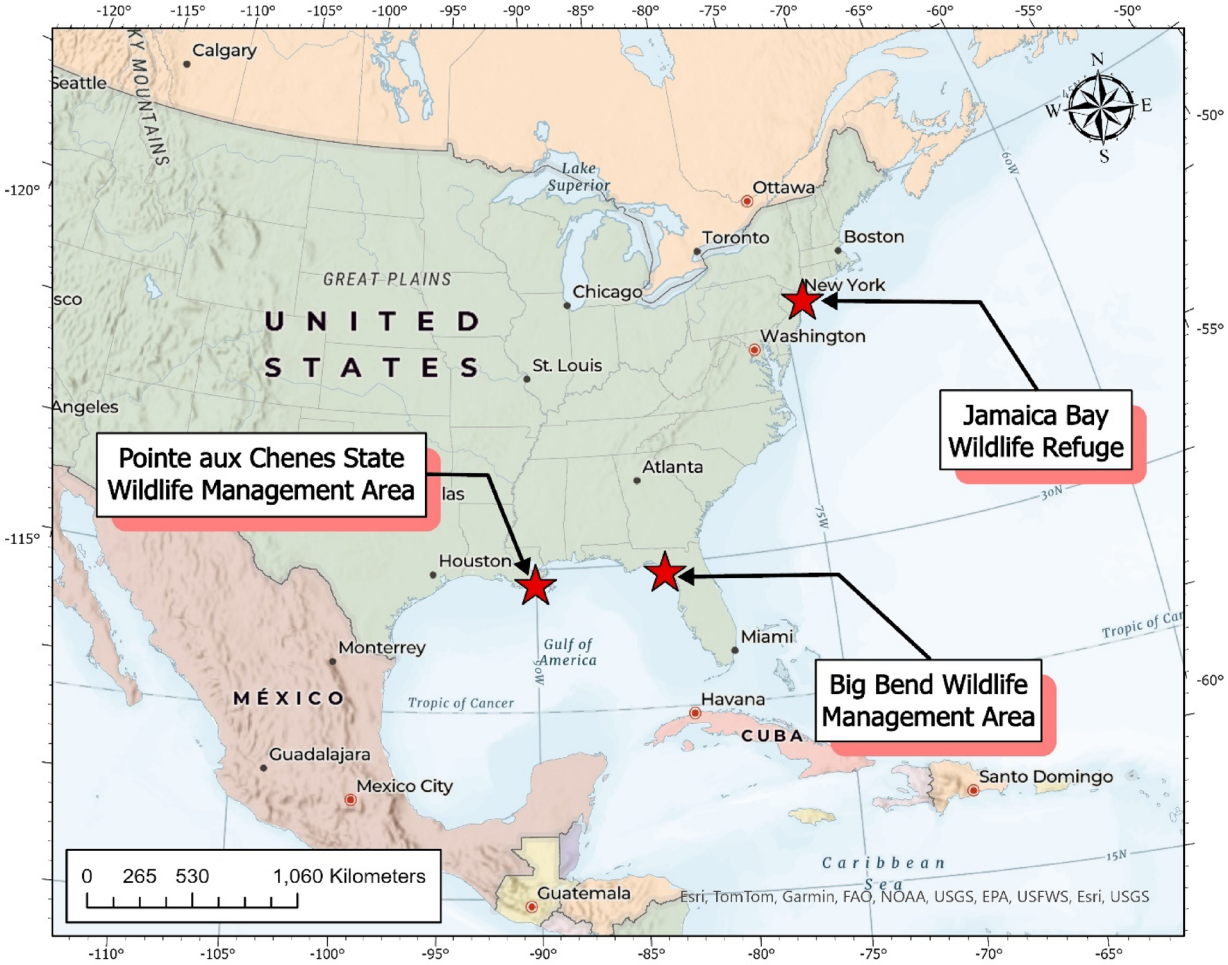
The locations of the three wildlife preserves in the United States used as test sites for final model selection are indicated with red stars. Map created in ArcGIS Pro (version 3.6.0, Esri; https://www.esri.com/) using the Charted Territory Map basemap.

The test dataset was created separately from the GSADB using high-resolution imagery. This imagery was obtained for three different wetlands located in wildlife preserves throughout the United States (Fig. 4). The high-resolution imagery for this exercise was PlanetScope imagery obtained from Planet Labs, with a spatial resolution of 3-meter. To match the methodology of the training data, GWL_FCS30D maps were used to help identify the wetland extent within the preserves, an NDVI map was calculated using the PlanetScope imagery, and the TerraSAR-X DEM was also utilized for each location. In addition, the PlanetScope imagery and the Sentinel-2 imagery were selected to ensure that their tidal levels close; differences in tidal level between the imagery sets were < 0.7 ft mean lower low water (MLLW). Sentinel-2 imagery from the same area as the PlanetScope imagery was secured through the GEE API. Only cloud free Sentinel-2 and PlanetScope imagery were used. In the case of the PlanetScope imagery, this was done by visual inspection. For the Sentinel-2 imagery, this was done by first filtering the Sentinel-2 imagery for a cloud cover of less than 30% and the remaining images were filtered using visual inspection.

Out of the 15 candidate models, five were selected for testing using the test dataset based on their performance during training/validation. Each of these five models was used to segment the Sentinel-2 imagery of wildlife preserves. The wetlands annotations created using the PlanetScope imagery were down sampled to 10-meter resolution to create a ground truth mask. The final accuracy metrics of OA, PA, UA, and IoU for each model were calculated by comparing the segmented images to these ground-truth masks. The model that achieved the highest accuracy metrics was then evaluated qualitatively. This was done by segmenting a large region around the annotation areas at each of the three test sites using the final model and visually inspecting the result. The workflow for model testing and final model selection are given in Fig. 1.

The Pointe aux Chenes State Wildlife Management Area served as the first test location. The wildlife preserve is in the Terrebonne Basin, an abandoned Mississippi River Delta in Louisiana. The preserve area is mostly salt marsh, with numerous ponds, bayous, sloughs, and canals. The marsh-bay-bayou complex is particularly notable as an important refuge for migratory birds, with reported sightings of over 300 species of birds^49^. The location used for segmentation is located slightly to the south of the preserve to avoid cloudy conditions in the imagery.

The Big Bend Wildlife Management Area of Florida near the Steinhatchee River was chosen as the second test location. The Big Bend region contains salt marshes that are characterized by tidal flooding. Further inland, the region exhibits large freshwater swamps, wet pinelands, and hardwood forests. These wetland habitats support marine fisheries as well as wildlife resources such as the Florida black bear^50^.

The Jamaica Bay Wildlife Refuge was selected for the third and final test location. Jamaica Bay is an estuary dotted with salt marsh islands. Given the preserve’s proximity to New York City, the marsh has experienced extensive anthropogenic influence. However, the area still serves as an important sanctuary for migrating birds. In addition, the marsh islands in the bay serve the city as a buffer against coastal storms^16,51^.

## Results

### Model training

**Fig. 5:**
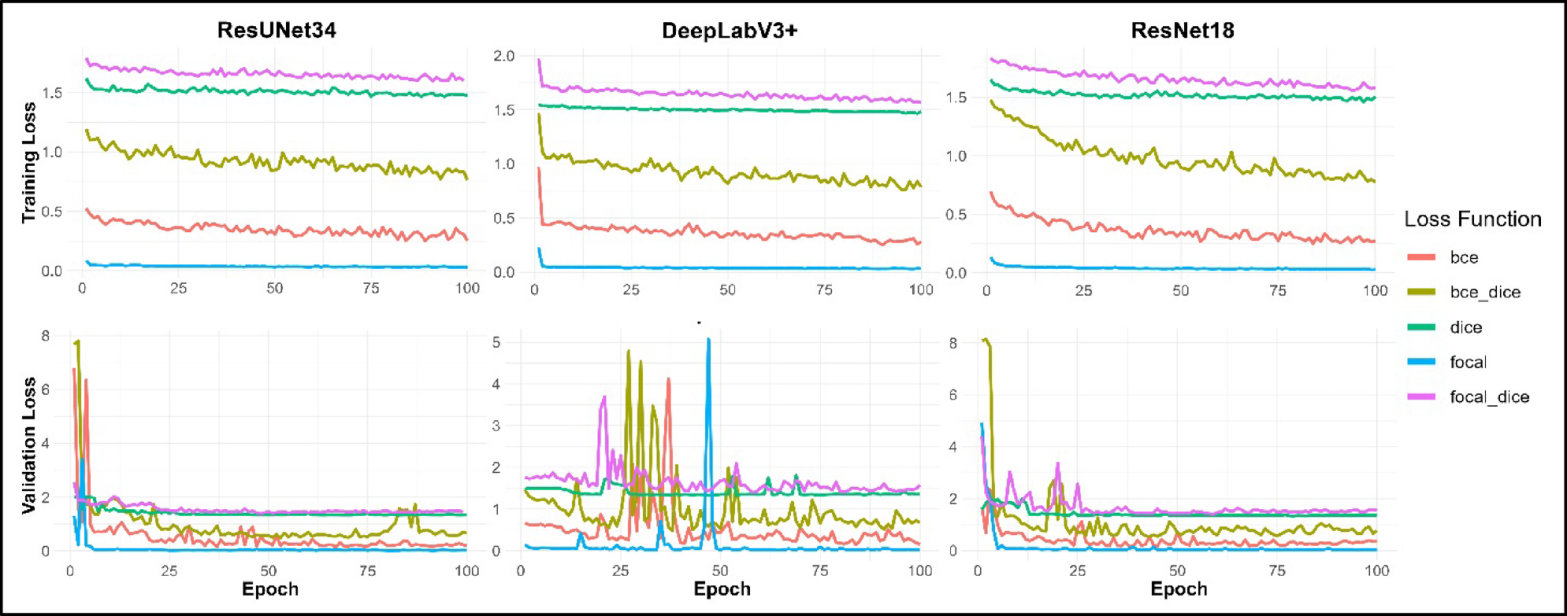
The training and validation set losses during model training plotted for all five of the loss functions across the three model architectures employed in this study.

All 15 models performed reasonably well during training, with both the training and validation losses largely converging over the 100 epochs. Although larger batch sizes can lead to smoother and more stable loss convergence, the batch size was constrained by GPU memory limitations. Within these constraints, the chosen batch size provided satisfactory training convergence. Similarly, the validation losses converged for most model runs but may also have benefited from a larger validation set. For DeepLabV3+, validation losses exhibited fluctuations towards the beginning of model training but mostly converge towards the end of the 100 training epochs (Fig. 5).

The accuracy metrics of the 15 candidate models indicate each model fitted reasonably well (Table S1). At the final epoch of training, the OA of the validation data ranged from 72% − 96%, while the IoU ranged from 55% − 93%. Given that loss functions and model architectures represent the primary variables in this analysis, Table 1 presents model performance averaged by loss function and by architecture to give a condensed view of the training results. The BCE loss function showed the highest average performance, with an averaged IoU and OA of 85% and 92%, respectively. In contrast, the focal loss function showed the lowest performance, with an averaged IoU and OA of 68% and 81%, respectively. Across architectures, ResUNet34 performed the best, with averaged scores of 81% IoU and 90% OA, compared to 78% IoU and 87% OA for DeepLabV3+, and 74% IoU and 85% OA for ResNet18.

**Table 1:** Averaged performance metrics of the candidate models on the final epoch of training, combined by either loss function or model architecture.

	Test category	Validation IoU	Validation OA	Training IoU	Training OA
Loss functions	BCE	0.85	0.92	0.77	0.89
	BCE + Dice	0.81	0.90	0.78	0.89
	Dice	0.78	0.88	0.61	0.77
	Focal + Dice	0.75	0.85	0.75	0.88
	Focal	0.68	0.81	0.70	0.86
Models	ResUNet34	0.81	0.90	0.72	0.86
	DeepLabV3+	0.78	0.87	0.71	0.85
	ResNet18	0.74	0.85	0.74	0.87

### Model selection

After training, the five candidate models with the highest OA and IoU on the validation set were selected for testing (Table S1). We used these models to segment wetlands in the three test locations: Jamaica Bay, Big Bend, and Pointe aux Chenes (Fig. 4). We then compared the segmentations with the high-resolution annotations to calculate their accuracy metrics to perform a final model selection. Ultimately, the ResUNet34 model paired with the focal-dice loss function performed the best with an averaged performance of 94%, 79%, 93% and 75% for OA, PA, UA, and IoU across the three test sites (Table 2).

**Table 2:** Averaged performance metrics of the top five candidate models across the three test site locations.

Model	Loss function	Overall accuracy	Producer’s accuracy	User’s accuracy	IoU
ResUNet34	Focal + Dice	0.94	0.79	0.93	0.75
DeepLabV3+	Dice	0.93	0.80	0.86	0.70
ResUNet34	BCE	0.92	0.70	0.95	0.67
ResUNet34	BCE + Dice	0.87	0.62	0.93	0.58
DeepLabV3+	BCE	0.83	0.45	0.91	0.42

**Fig. 6:**
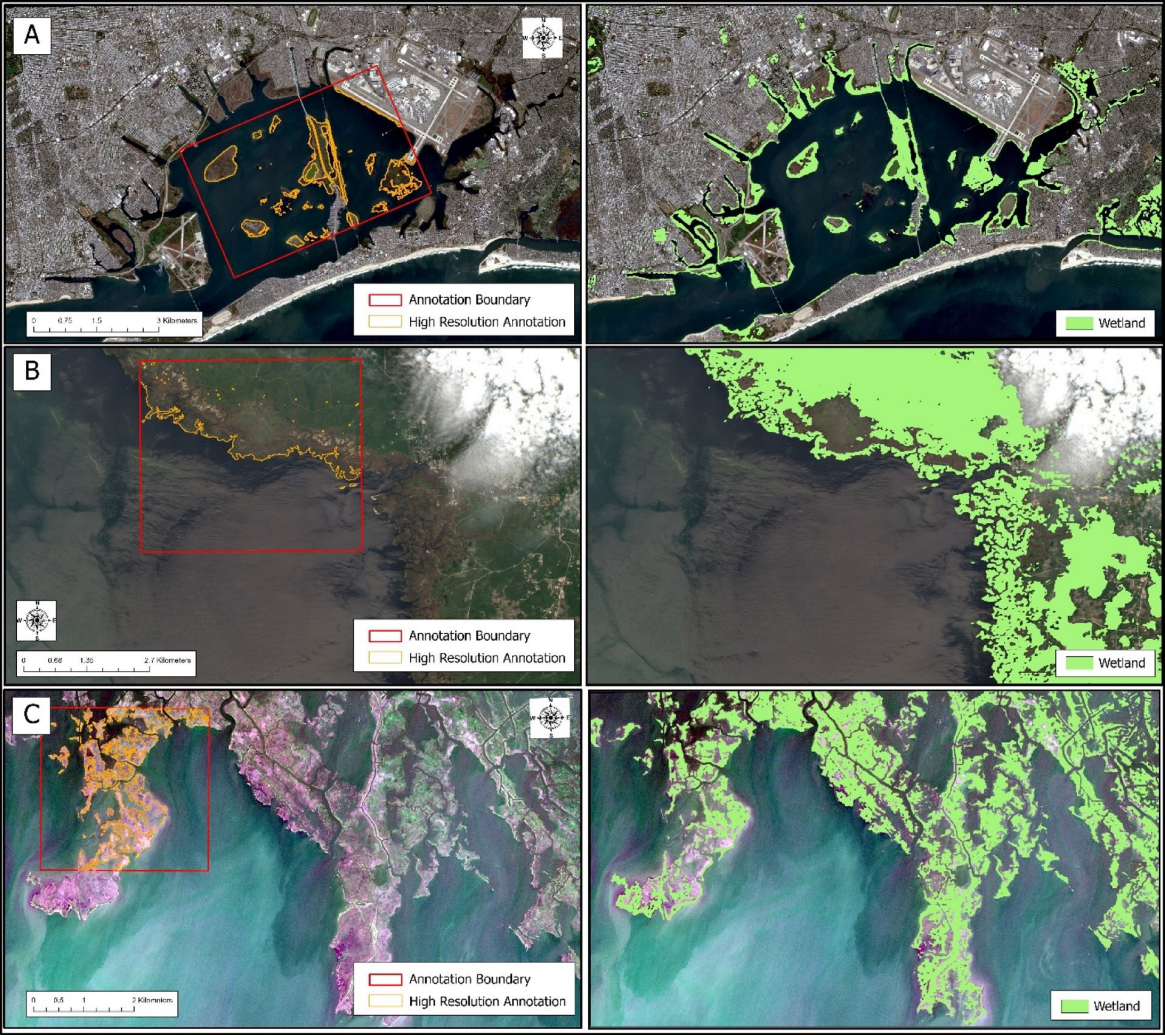
A comparison of the PlanetScope hand annotations and Swamp-AI classifications of Sentinel-2 imagery with the segmented images of Jamaica Bay (**A**), the Big Bend region (**B**), and south of Pointe aux Chenes (**C**). The first column shows PlanetScope hand annotations. The exact locations used for model validation are illustrated by the red bounding boxes and the annotations delineating the wetlands within are shown in orange for reference. The second column shows the Swamp-AI classifications in green. For the purposes of illustration, the non-wetlands class has been masked. Satellite imagery was obtained from Google Earth Engine using the Harmonized Sentinel-2 MSI Level-2 A Surface Reflectance dataset (COPERNICUS/S2_SR_HARMONIZED), and processed within ArcGIS Pro (version 3.6.0, Esri; https://www.esri.com/).

As a final check, we performed a qualitative analysis of the ResUNet34 paired with focal-dice model. Examination of the segmented maps revealed this model demonstrated reliable performance outside the annotation area at each of the three test sites (Fig. 6). Therefore, the ResUNet34 architecture, paired with the focal-dice loss function, was selected as the final model for Swamp-AI.

## Discussion

### Swamp-AI: a wetlands segmentation model

The primary objective of Swamp-AI was to develop a model capable of segmenting wetlands anywhere. Many wetland classification modeling studies have focused on multi-class classification, aiming to segment classes such as plant species, local types of wetlands, and man-made developments found in the target area^11,14,18,19,23,29^. While such approaches provide fine-grained detail, they are typically constrained to specific sites or regions. In contrast, Swamp-AI is a binary classifier that segments images into wetland and non-wetland classes. This simplification was necessary to enhance Swamp-AI’s generalizability for several reasons. First, the GSADB data was annotated without direct field validation. Second, multi-class segmentation introduces complexity into model training, as the model’s loss function favors optimization of the dominant classes^46^. The strong performance of Swamp-AI on the three independent test sites further validates the choice of Swamp-AI as a binary classifier for a global-scale wetland segmentation model.

Comparison of Swamp-AI with other DL wetland segmentation models further serves to underscores Swamp-AI’s potential. For example, the use of DEMs and topographic maps to annotate wetland areas has been performed in numerous other studies^14,18,22,23^. In terms of accuracy, Swamp-AI demonstrates comparable levels of accuracy to other ML and DL wetland segmentation studies, boasting an OA of 94% and an IoU of 74% with two classes (i.e., wetland and non-wetland). For example, the GWL_FCS30D wetland landcover maps achieved 86.4% OA with 8 wetlands classes world-wide^9,10^. While the OA of Swamp-AI is slightly higher, Swamp-AI only segments two classes and therefore its classification task is less complex. In a different study, Dang et al. (2020) employed a similar model architecture to Swamp-AI in the Tien Yen Estuary of Vietnam and achieved a final score of 90% OA and 83% IoU over 10 wetland classes. While Swamp-AI’s IoU is slightly lower than this regional scale model, mapping wetlands on a global-scale presents challenges distinct from those in a specific locality^15^. That Swamp-AI is similar in accuracy to regional scale models serves to further emphasize Swamp-AI’s potential as a wetland segmentation model (Table 3).

**Table 3:** Comparison of Swamp-AI with contemporary DL and ML wetland segmentation models. For the sake of comparison, the accuracy listed for^29^ is taken from the model runs using Sentinel-2 data.

Source	Locations	Overall accuracy (%)	Wetlands classes
Swamp-AI	Global	94	2
Zhang^9^	Global	87	8
Pham^13^	Tien Yen and Ba Lat Estuaries	86	13
Dang^18^	Tien Yen Estuary	90	10
Liu^29^	Honghe National Nature Reserve	96	7
Ke^19^	Liahoe Estuary	94	6
Jiang^23^	Northwest Territories, Canada	93	6

### Final model selection

Model selection for Swamp-AI focused on testing combinations of loss functions and model architectures to identify which combination would be best suited to model the GSADB. The primary considerations for selecting the model architectures and loss functions were the number of training images in the GSADB and the class balance in the annotated images. The GSADB comprises a total of 102 images, a relatively small dataset for a DL exercise. However, the DeepLabV3 + model was successfully employed in mapping surface water using 95 annotated images^33^, while the ResUNet34 model is based on the U-Net architecture which was designed to handle their a dataset of only 30 biomedical images^21^. To address class balance, we intentionally selected images that contained various levels of wetlands and non-wetlands so the final model would generalize well across wetland and non-wetland areas. Both the dice and focal loss functions are known to perform well with imbalanced datasets^18,24,46,52^. Finally, while the cross-entropy loss function is not typically used under class imbalance, it was included as a baseline since it is a widely used loss function.

Of the three model architectures, the ResUNet34 model performed the best (Tables 1 and 2). The disparity in performance between ResNet18 and ResUNet34 is particularly notable given their similarity in model architecture. Namely, both models rely on stacking residual blocks in the encoder portion of the model. The most significant differences between these two models being the number of trainable parameters and the use of a concatenate operation. ResUNet34 comprises many more trainable parameters than the ResNet18 model, with the ResNet18 model comprising ~ 13 million trainable parameters compared to ~ 24 million trainable parameters for ResUNet34. The increased number of trainable parameters may have given the ResUNet34 model an advantage over the ResNet18 model. Also, the ResUNet34 architecture includes a concatenate operation, which creates the symmetrical contracting-expanding model path that U-Net is known for.

The difference between DeepLabV3 + and ResUNet34 was also unexpected. Unlike the ResNet18 model, the DeepLabV3 + model comprises many more trainable parameters than the ResUNet34 model; the DeepLabV3 + model used here contains ~ 54 million trainable parameters, compared to the ~ 24 million trainable parameters of the ResUNet34 model. Given the relatively small size of the GSADB, with only 102 images, it is possible that the increased number of parameters may have caused the larger DeepLabV3 + model to overfit to the training data.

We believe that the ResUNet34 model’s superior performance likely derives from the symmetrical architecture of U-Net. The original U-Net model was designed to handle very small datasets by performing both data augmentation and employing a symmetric contracting-expanding model architecture^21^. While the use of data augmentation was not unique to the ResUNet34 models, the symmetric U-Net structure (i.e., the concatenate operations) likely allowed the ResUNet34 models to better fit a small database and thus outperform the other two candidate model architectures.

Of the loss functions tested, the dice loss and focal-dice loss functions performed the best, while the focal loss function alone performed very poorly. The focal loss function was tuned to deemphasize the wetlands class and to emphasize the non-wetlands class^46^. As a result, the focal loss’ criteria for identifying wetlands proved too strict. In contrast, while the dice loss functions demonstrated high accuracy metrics (Table 2), visual examination of the segmented maps created by the dice loss models revealed that these models’ criteria for identifying wetlands was too relaxed (Fig. S1). The focal-dice loss function achieved the highest accuracy metrics, suggesting that the combination of these two criteria extremes struck an effective balance. The advantages of combining these two loss functions have been reported by other studies^18^. For the focal loss function to perform well on the GSADB as a stand-alone loss function, it would need to be reconfigured to emphasize the wetlands class and deemphasize the non-wetlands class.

### Jamaica Bay case study: time series analysis

An advantage of Swamp-AI is its ability to track wetland extent throughout the world, including the wildlife preserves examined in this study (Fig. 6). To demonstrate an application of Swamp-AI, we performed a case study focused on the salt marsh islands of the Jamaica Bay Wildlife Refuge. In this case study, we used Swamp-AI as a time series analysis tool for detecting wetlands under stress in Jamaica Bay. These islands are of particular interest due to concerns that their wetlands are under stress^16,53–55^. Consequently, significant restoration efforts have been undertaken.

**Fig. 7:**
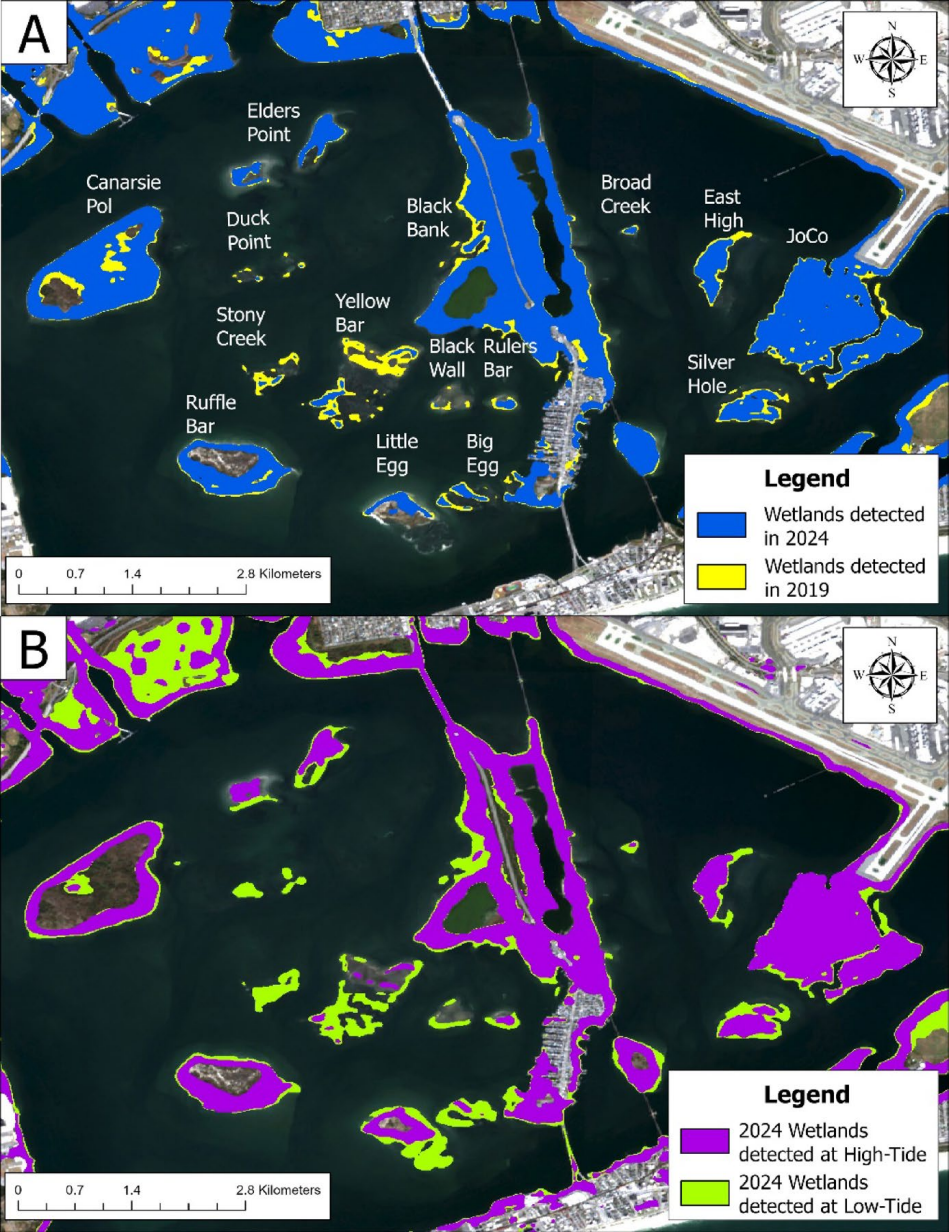
Wetlands classification of Jamaica Bay. Shown here are the classifications run using the 2019 and 2024 Sentinel-2 annual composites (**A**) and the classifications run using the 2024 high- and low-tide Sentinel-2 images (**B**). Satellite imagery was obtained from Google Earth Engine using the Harmonized Sentinel-2 MSI Level-2 A Surface Reflectance dataset (COPERNICUS/S2_SR_HARMONIZED), and processed within ArcGIS Pro (version 3.6.0, Esri; https://www.esri.com/).

For the use-case analysis, we performed two classification exercises of Jamaica Bay, one using Sentinel-2 annual composites for the years 2019–2024, and the other using two images taken in 2024 representing high- and low-tide scenes of the bay. The three scenarios of high-tide, low-tide and annual composite (i.e., mean sea level) were chosen because Swamp-AI was trained to classify inundated wetlands as non-wetlands. Given that some wetlands are inundated based on tidal level, Swamp-AI’s predictions should vary accordingly. Ultimately, we anticipated that Swamp-AI would identify the most wetland area at low tide, when inundation is minimal, and less at mean sea level or high tide. All images for this exercise were obtained and processed through the GEE API. For the annual composites analysis, each annual composite image was generated using the median of cloud-free Sentinel-2 imagery taken over the course of the corresponding year. Annual composites were used to provide a simplified, representative view of the bay for that year. Furthermore, the use of median annual composites effectively removes the influence of tidal level from the imagery, particularly in high-turbidity wetlands^56^. To make the composites cloud-free, the imagery was filtered for a cloud cover of less than 30% and a bitwise mask for cloud pixels was applied. These annual composites were then segmented using Swamp-AI, which maps were used to calculate the extent of the wetlands on each island from year to year. For the tidal analysis, an image taken January 2, at 2.9 ft MLLW was used as the high-tide image and an image was taken February 6, at 0.25 ft MLLW was used as the low-tide image. The tidal level was measured by a nearby NOAA tide gauge: #8531680 Sandy Hook, New Jersey. Both images were classified by Swamp-AI and compared to demonstrate the effect of tidal level on Swamp-AI’s classifications. Figure 7 provides a condensed view of the analysis, illustrating the annual composite wetlands areas detected in 2019 and 2024 as well as the wetlands identified at high- vs. low-tide in 2024.

**Fig. 8:**
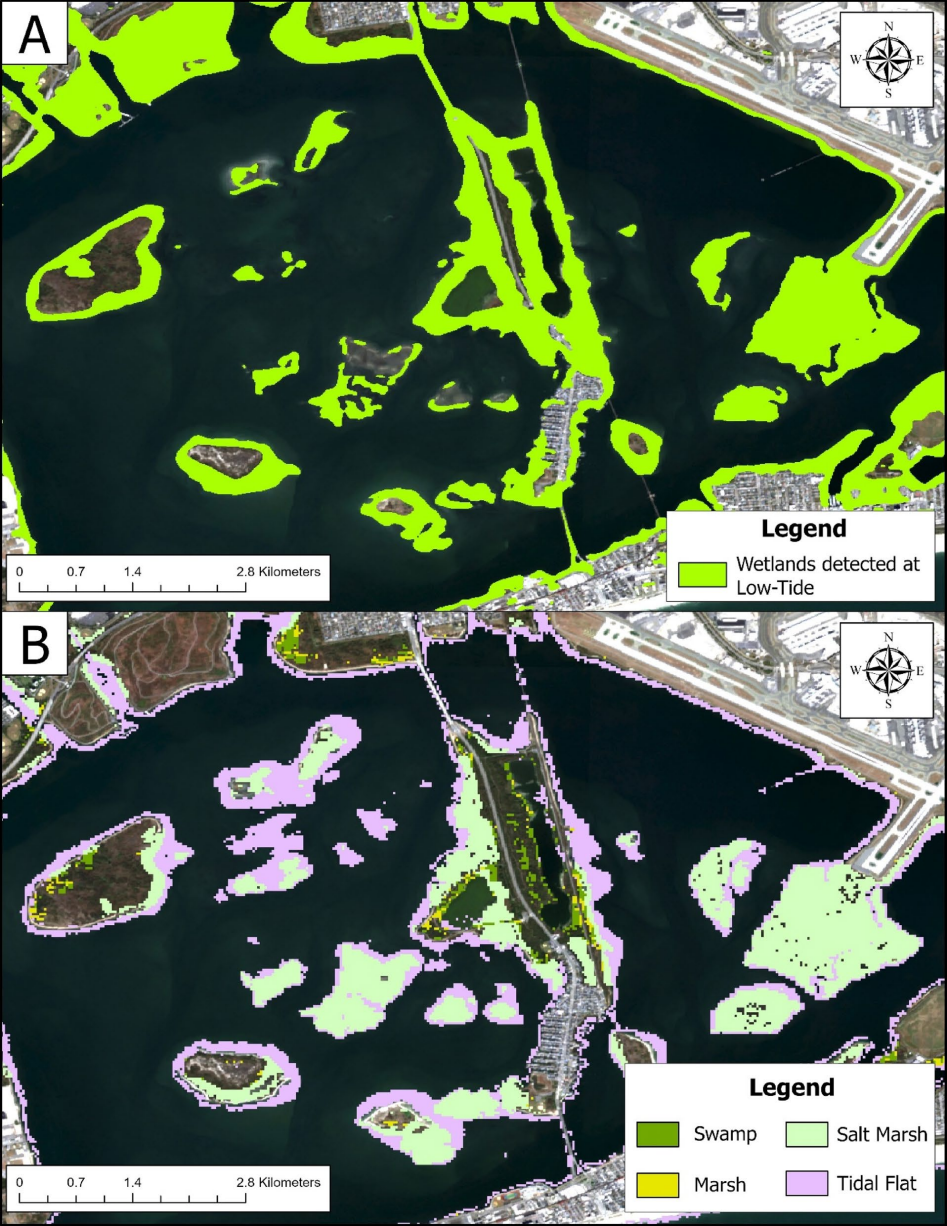
Comparison of wetlands classifications in Jamaica Bay, NY, as performed by Swamp-AI using a low-tide Sentinel-2 image (**A**) and the 2019 GWL_FCS30D map (**B**). Satellite imagery was obtained from Google Earth Engine using the Harmonized Sentinel-2 MSI Level-2 A Surface Reflectance dataset (COPERNICUS/S2_SR_HARMONIZED), and processed within ArcGIS Pro (version 3.6.0, Esri; https://www.esri.com/).

Deploying Swamp-AI on the salt marshes of Jamaica Bay revealed that the extent of these marshes is still declining (Fig. 7A). From the annual composite analysis, we calculated an average decline of 1.32 ha yr^− 1^ (standard error = 0.38 ha yr⁻¹) of wetland from each island. However, comparison of the 2024 annual composite, high-tide, and low-tide analyses reveals that each analysis provides slightly different classifications of the wetlands. Except for Canarsie Pol, the high-tide classification is similar to the annual composite prediction, with Swamp-AI identifying a total of 1,489 ha of wetland in the composite scene and 1,407 ha in the high-tide scene. In contrast, the low-tide scene identified 1,884 ha of wetland, much more than either the annual composite or high-tide scenes. However, the low-tide classification demonstrates superior agreement with the GWL_FCS30D maps (Fig. 8). These results illustrate how sensitive Swamp-AI’s classifications are with respect to tidal level. The low-tide scenario represents a scene where tidally inundated wetlands are visible to Swamp-AI. In contrast, the high-tide scenario represents a more minimal prediction, since parts of the wetland are entirely inundated at high tide and ultimately classified as non-wetland. This presents a trade-off for future analysis. While using annual composites offers a straightforward method for time-series analysis, it presents the model with composite imagery that differs from its training data. On the other hand, although the tidal analysis was purely for qualitative comparison, it demonstrates that incorporating imagery from various tidal levels can yield more nuanced and valuable information about wetland extent.

According to the annual composites analysis, the total loss of wetlands for the five-year period was estimated at 18.4 ha yr^− 1^, with a standard error of 0.57 ha yr^− 1^ (R^2^ = 0.89). To estimate wetlands loss, we fit a line to the area of detected wetlands from each year over the five-year period and used its slope as the rate of change (Fig. S2). Here, the R^2^ refers to the consistency of change detected by Swamp-AI, with a low R^2^ indicating no or inconsistent change and a high R^2^ indicating consistent change over this period. The decline of these wetlands is well documented; one study estimated that from 1989 to 2003 approximately 13.4 ha were lost annually^53^. Another study estimated that from 2003 to 2013 approximately 2.1 ha of wetlands were lost annually^16^. More recently, a study investigating sediment transport into Jamaica Bay found that while net sediment import into the bay is positive, the current rate of supply is not sufficient to maintain the islands relative to sea-level rise^57^.

Salt marsh decline is not constant across the different islands of Jamaica Bay, therefore the annual change in wetland coverage for each island was estimated using the same approach as above. In addition, the results from the annual composite analysis suggest that some of the islands are stable, while others are under stress. For example, Little Egg shows little evidence of change to its wetlands while Yellow Bar shows more significant indicators of wetland decline during this time (Table 4). Percent change is relative to the size of the island in 2019.

**Table 4:** The predicted annual wetlands landcover change for each of the salt marsh Islands in Jamaica Bay, NY based on a time series analysis between 2019 and 2024.

Island	Calculated annual change (ha)	Calculated annual change (%)	*R* ^2^	Standard error
JoCo	-2.76	-1.50	0.96	1.16
Yellow Bar	-5.28	-17.19	0.96	2.39
East High	-1.17	-4.87	0.93	0.67
Silver Hole	-1.21	-5.01	0.88	0.93
Stony Creek	-1.36	-18.30	0.88	1.05
Black & Ruler Bar	-0.63	-12.75	0.86	0.54
Broad Creek	-0.12	-8.23	0.85	0.11
Duck Point	-0.44	-21.72	0.81	0.44
Canarsie Pol	-2.73	-2.13	0.51	5.55
Elder Point	-0.56	-2.72	0.34	1.64
Black Bank	-1.43	-1.18	0.24	5.30
Big Egg	-0.40	-1.82	0.14	2.02
Ruffle Bar	-0.38	-0.87	0.13	2.07
Little Egg	0.03	0.43	0.00	1.67

### Wetlands of the world case study

To demonstrate Swamp-AI’s global application, we applied the model to classify seven well-known wetlands located throughout the world. Swamp-AI was trained and validated on globally distributed scenes. New scenes representing diverse wetland systems that Swamp-AI has never seen before are used to demonstrate Swamp-AI’s capabilities to classify wetlands throughout the world. To quantify Swamp-AI’s accuracy on these scenes, we annotated the imagery using the same methodology used to annotate the GSADB images (Figs. S3–S9).

We examined the effect of location on Swamp-AI’s performance. Swamp-AI’s segmentation was compared to the hand-drawn annotations to quantify its performance (Table 5). In addition, we also examined the effect of adjusting Swamp-AI’s probability threshold for classifying wetland pixels. The Swamp-AI model returns a probability (α) for each pixel, indicating the model’s confidence that a pixel is a wetland pixel. Throughout this study, we have employed a constant probability threshold of α = 0.5. However, we found that the optimal threshold might vary depending on a wetland’s climate.

To determine each wetland’s climate, we used the high-resolution (1 km) Köppen-Geiger maps for 1990–2020^58^. Except for the Al-Sudd wetland, Swamp-AI demonstrated superior performance using a higher probability threshold (α > 0.4) when segmenting wetlands from tropical climate regimes. In contrast, Swamp-AI typically preferred a lower probability threshold (α < 0.4) when segmenting wetlands from non-tropical climate zones (cold, temperate or arid).

**Table 5:** A qualitative analysis of Swamp-AI’s performance on wetlands across the world. The climate label was determined using the Köppen-Geiger classification. The ideal α refers to the wetland probability threshold that produced the highest overall accuracy.

Name	Country	Accuracy (ideal α)	IoU (ideal α)	Ideal α	Climate
Hudson Bay	Canada	0.85	0.76	0.1	Cold
Al-Sudd	South Sudan	0.91	0.49	0.2	Tropical
Oakavango Delta	Botswana	0.98	0.98	0.2	Arid
Camargue	France	0.92	0.71	0.2	Temperate
Mamukala Wetlands	Australia	0.89	0.69	0.4	Tropical
Pantanal	Brazil	0.86	0.81	0.6	Tropical
Mekong Delta	Vietnam	0.92	0.82	0.9	Tropical
Average	-	0.91	0.75	-	-

When using the ideal probability threshold for each location, Swamp-AI demonstrated average accuracy and IoU values of 90.5% and 75.1%, respectively. These values are consistent with the previous estimates on the high-resolution test images, where the overall accuracy and IoU values were 93.7% and 74.6%, respectively. It is important to note that the probability threshold was held at a fixed α = 0.5 for all other evaluations of Swamp-AI’s performance for the sake of consistency. The probability threshold was studied here specifically as part of examining Swamp-AI’s performance on diverse wetlands globally. Consequently, we recommend using a higher probability threshold (α > 0.4) for wetlands in tropical areas, where Swamp-AI appears to over-classify vegetation as wetlands, and a lower threshold (α < 0.4) in non-tropical areas where Swamp-AI appears to detect wetlands yet assign them a lower confidence. This adaptability reinforces Swamp-AI’s potential as a versatile and promising tool for global wetland monitoring.

As sea level rises and extreme weather events threaten wetland ecosystems, efficient identification tools will prove increasingly vital. Swamp-AI, though limited to a single wetland class, showed reliable performance across diverse regions globally. We submit that Swamp-AI’s performance demonstrates its potential to support large-scale wetland monitoring and advise on conservation priorities.

## Supplementary Information

Below is the link to the electronic supplementary material.

Supplementary Material 1

## Data Availability

All code, imagery and annotated images described in this work are publicly available on github at: [https://github.com/candros/GSADB](https:/github.com/candros/GSADB) .
