# Supplementary material for: Swamp-AI: a deep learning model for monitoring wetlands change across the globe
Source: Sci Rep. 2026 Feb 13;16:8830. doi: 10.1038/s41598-026-39257-1 (PMC12982673; doi:10.1038/s41598-026-39257-1)
Supplement: Supplementary file 1 — Supplementary Material 1 [file 41598_2026_39257_MOESM1_ESM.docx]

# Supplementary Materials


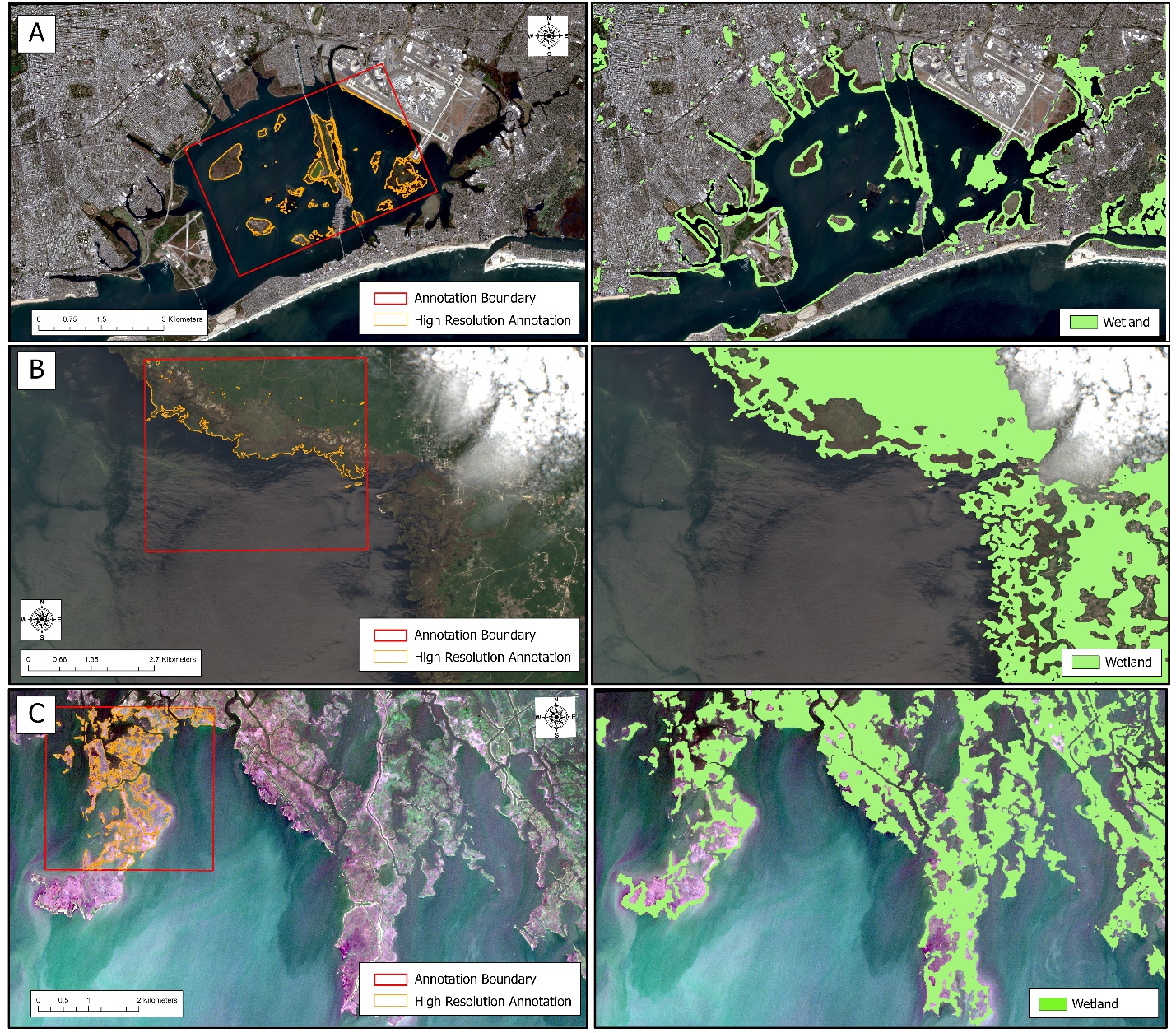


Figure S1. A comparison of the Sentinel-2 imagery with the segmented images of Jamaica Bay (A), the Big Bend region (B), and south of Pointe aux Chenes (C) using the DeepLabV3+ paired with the dice loss function. Note how the model overestimates the wetland extent compared to the ResUNet34 model, particularly in the urban regions. For the purposes of illustration, the non-wetlands class has been masked. Satellite imagery was obtained from Google Earth Engine using the Harmonized Sentinel-2 MSI Level-2A Surface Reflectance dataset (COPERNICUS/S2_SR_HARMONIZED), and processed within ArcGIS Pro (version 3.6.0, Esri; <https://www.esri.com/>).


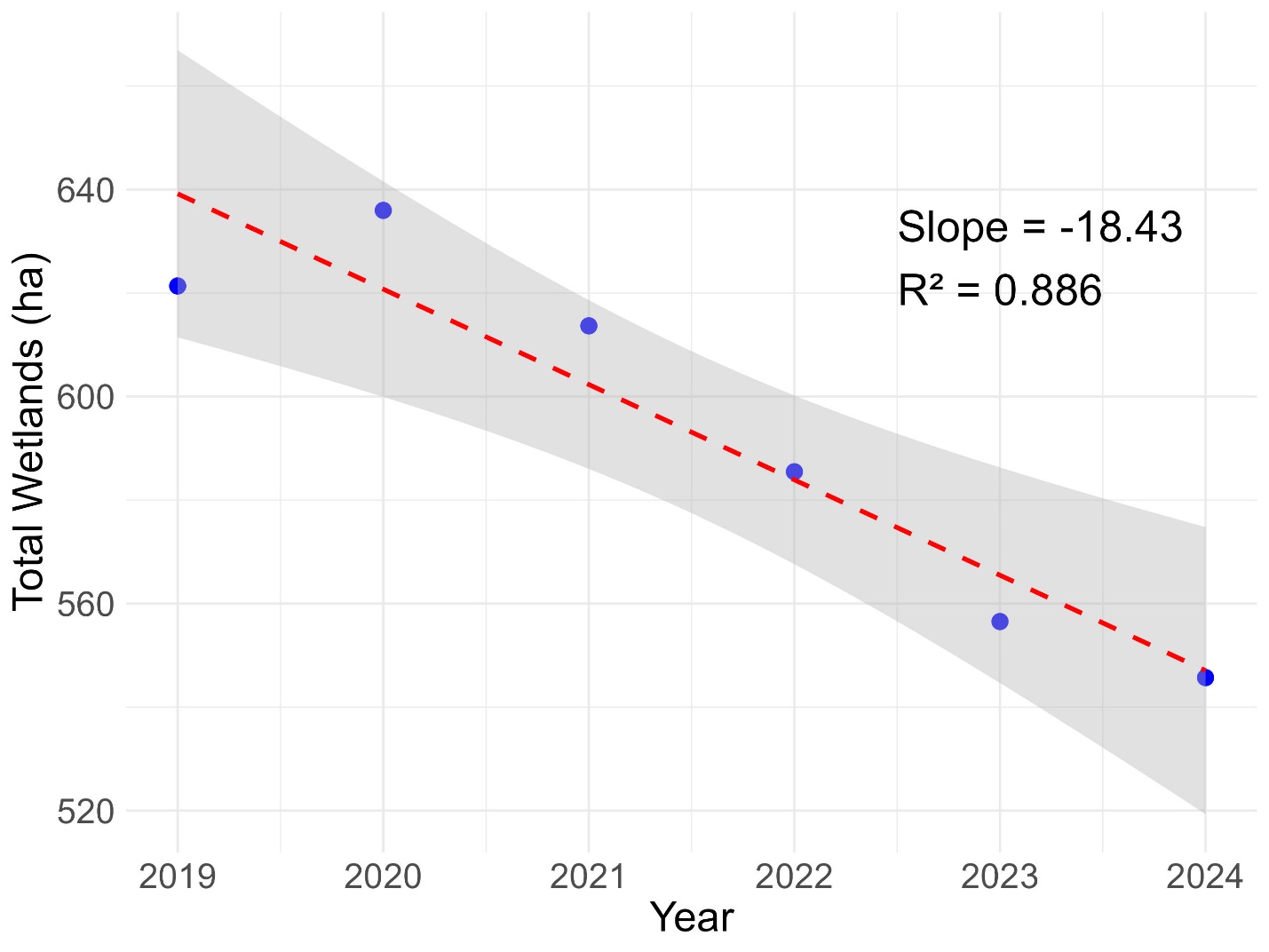

Figure S2. Plot of wetland areas detected by Swamp-Eye for all islands in Jamaica Bay in the five-year period of 2019 – 2024. Shown in red is the line of best fit with the 95% confidence interval. The slope of this line was used to estimate annual change in the extent of these wetlands.


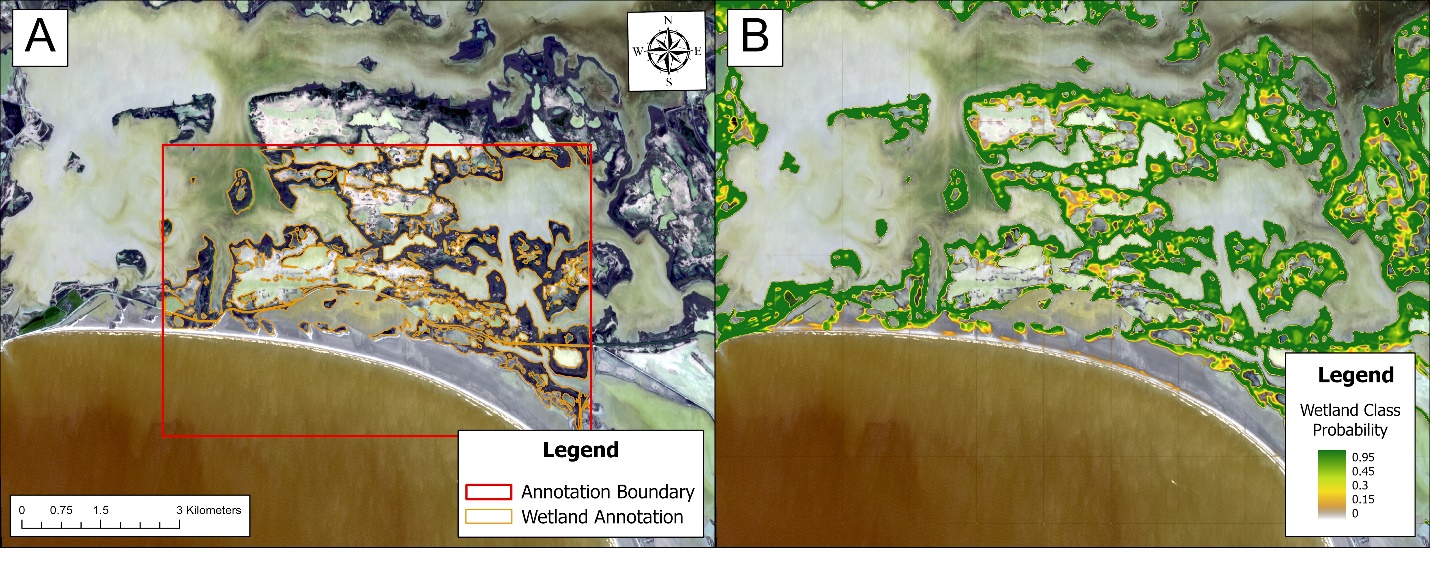


Figure S3. Annotated (A) and Swamp-Eye segmented (B) images of the Camargue Regional Nature Park, France. Image was taken 4/13/2017 near 4.5°E, 43.4°N. Satellite imagery was obtained from Google Earth Engine using the Harmonized Sentinel-2 MSI Level-2A Surface Reflectance dataset (COPERNICUS/S2_SR_HARMONIZED), and processed within ArcGIS Pro (version 3.6.0, Esri; <https://www.esri.com/>).


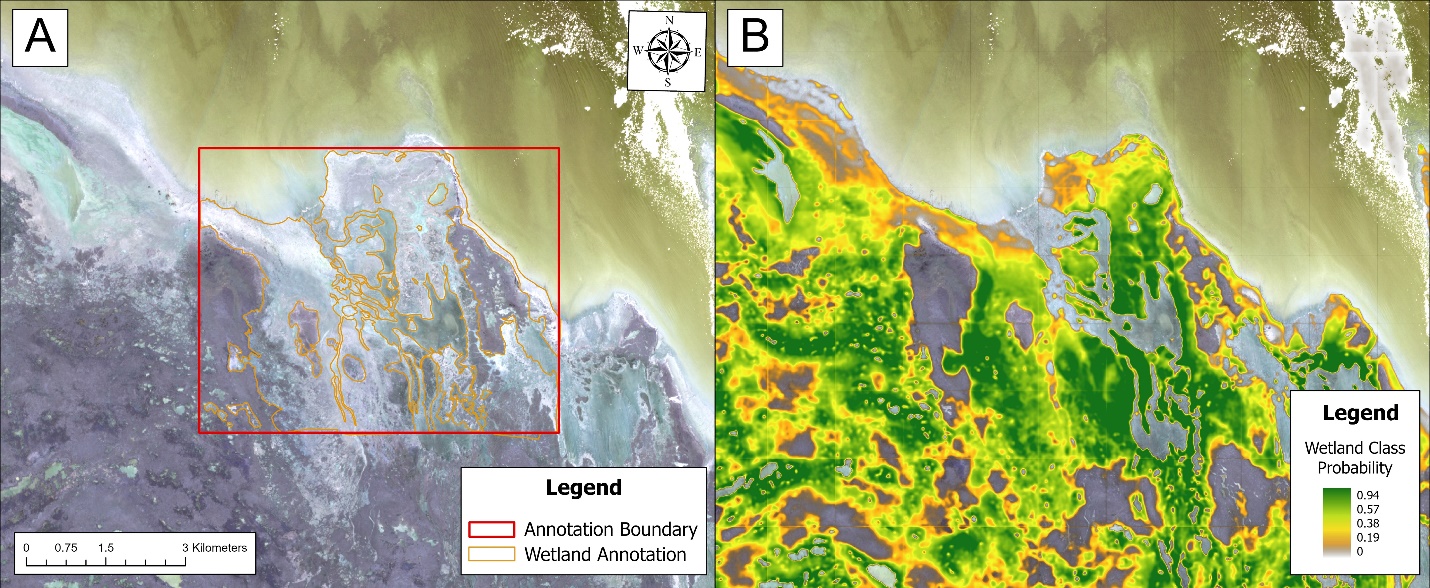


Figure S4. Annotated (A) and Swamp-Eye segmented (B) images of Hudson Bay, Canada. Image was taken 6/23/2018 near 82.5°W, 55.1°N. Satellite imagery was obtained from Google Earth Engine using the Harmonized Sentinel-2 MSI Level-2A Surface Reflectance dataset (COPERNICUS/S2_SR_HARMONIZED), and processed within ArcGIS Pro (version 3.6.0, Esri; <https://www.esri.com/>).


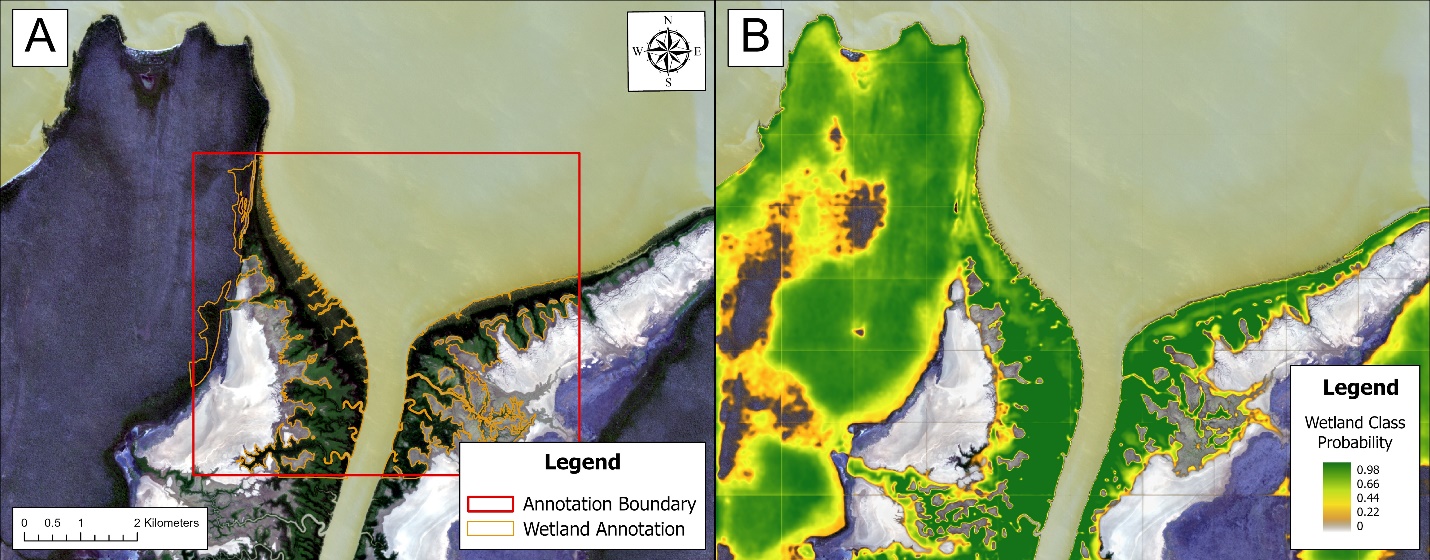


Figure S5. Annotated (A) and Swamp-Eye segmented (B) images of the Mamukala Wetlands, Australia. Image was taken 8/25/2017 near 132.2°E, 12.2°S. Satellite imagery was obtained from Google Earth Engine using the Harmonized Sentinel-2 MSI Level-2A Surface Reflectance dataset (COPERNICUS/S2_SR_HARMONIZED), and processed within ArcGIS Pro (version 3.6.0, Esri; <https://www.esri.com/>).


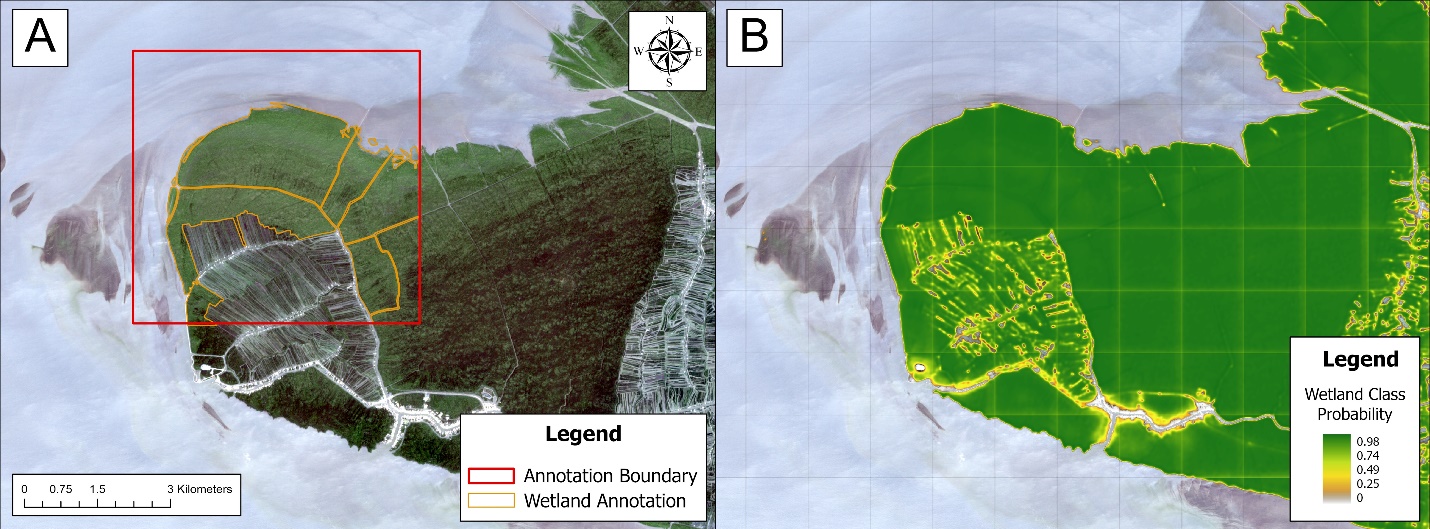


Figure S6. Annotated (A) and Swamp-Eye segmented (B) images of the Mekong Delta, Vietnam. Image was taken 2/20/2020 near 104.7°E, 8.6°N. Satellite imagery was obtained from Google Earth Engine using the Harmonized Sentinel-2 MSI Level-2A Surface Reflectance dataset (COPERNICUS/S2_SR_HARMONIZED), and processed within ArcGIS Pro (version 3.6.0, Esri; <https://www.esri.com/>).


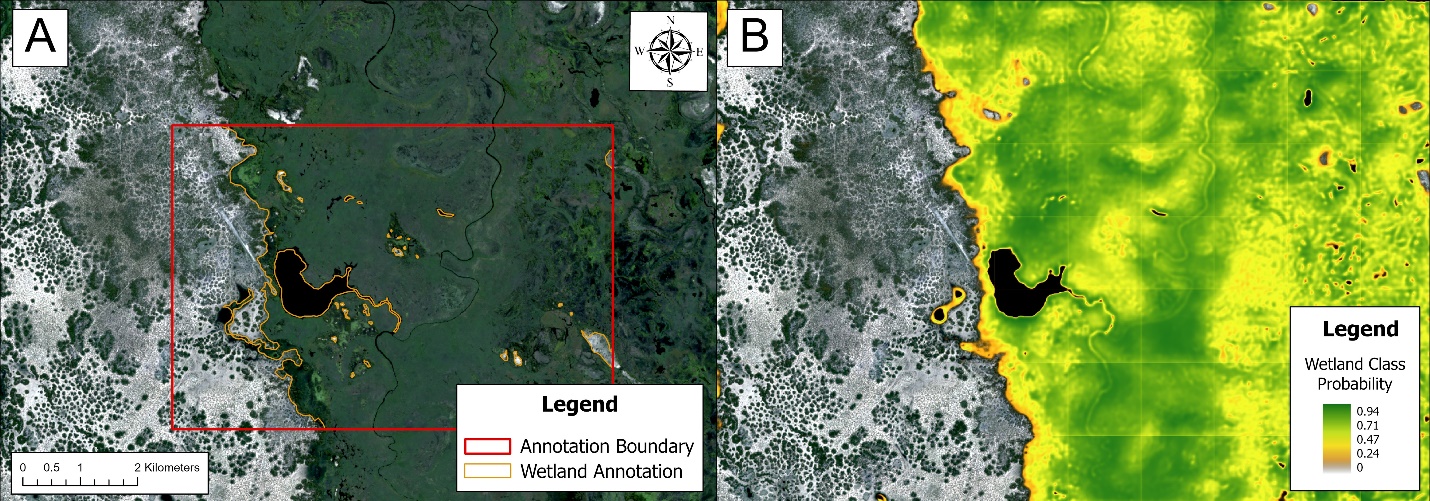


Figure S7. Annotated (A) and Swamp-Eye segmented (B) images of the Oakavango Delta, Botswana. Image was taken 4/11/2017 near 22.3°E, 18.9°S. Satellite imagery was obtained from Google Earth Engine using the Harmonized Sentinel-2 MSI Level-2A Surface Reflectance dataset (COPERNICUS/S2_SR_HARMONIZED), and processed within ArcGIS Pro (version 3.6.0, Esri; <https://www.esri.com/>).


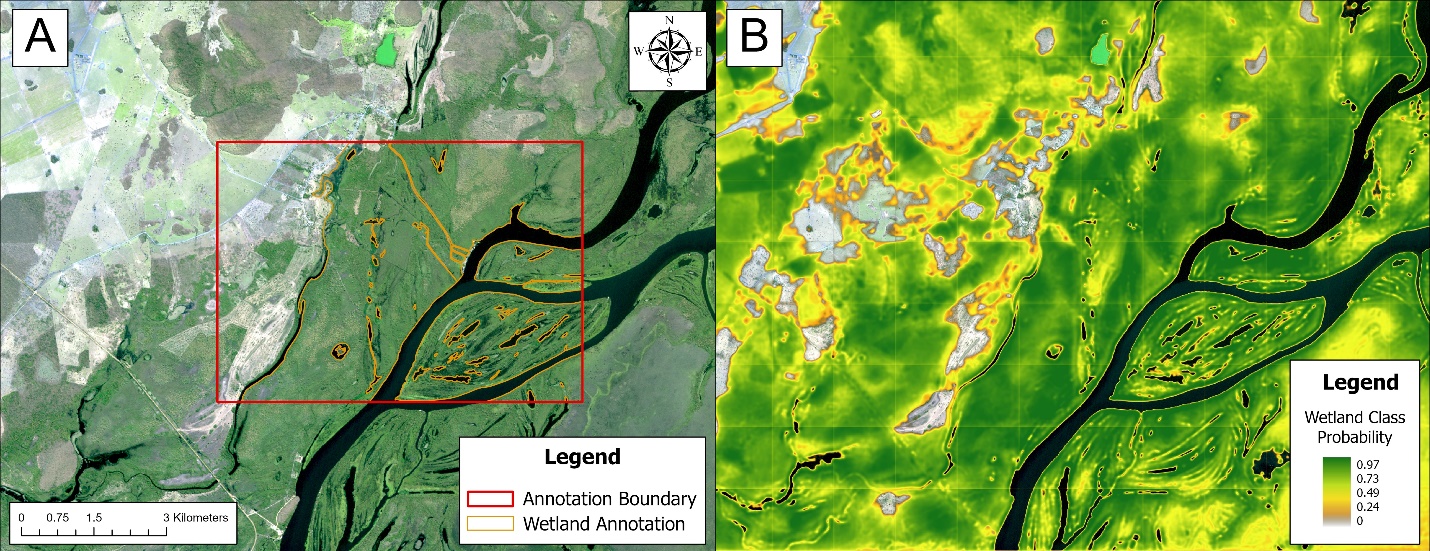


Figure S8. Annotated (A) and Swamp-Eye segmented (B) images of the Pantanal, Brazil. Image was taken 9/19/2017 near 57.3°W, 19.4°S. Satellite imagery was obtained from Google Earth Engine using the Harmonized Sentinel-2 MSI Level-2A Surface Reflectance dataset (COPERNICUS/S2_SR_HARMONIZED), and processed within ArcGIS Pro (version 3.6.0, Esri; <https://www.esri.com/>).


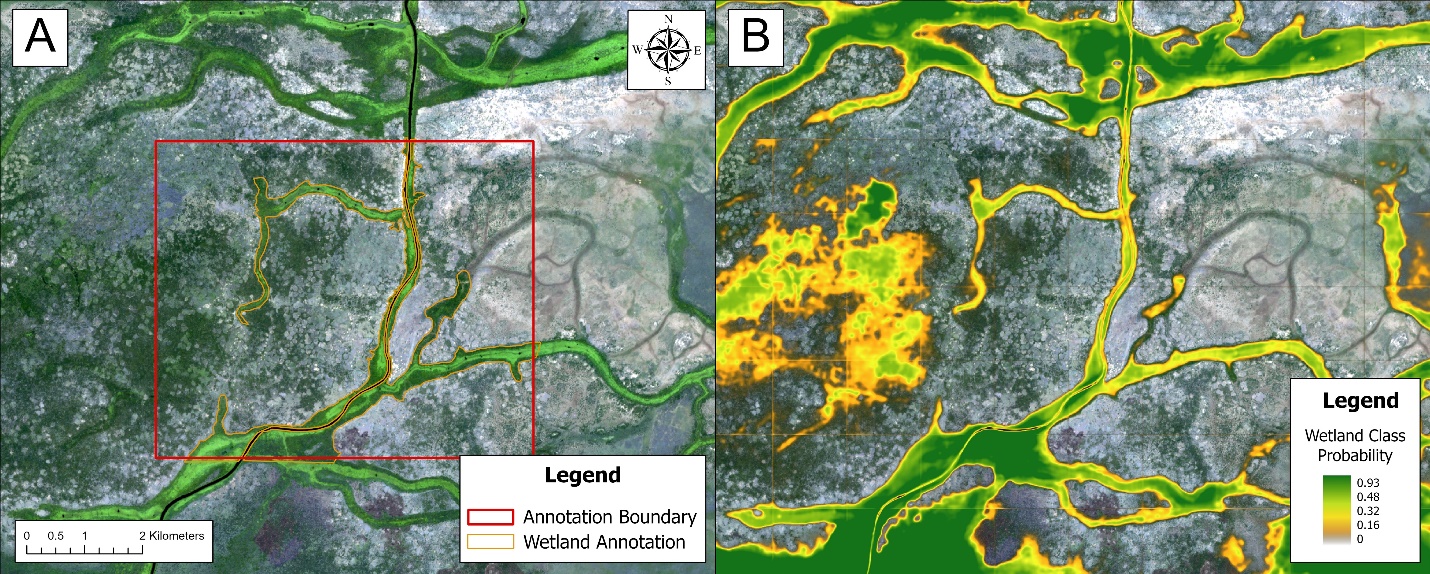


Figure S9. Annotated (A) and Swamp-Eye segmented (B) images of Al-Sudd, South Sudan. Image was taken 11/9/2018 near 31.1°E, 9.3°N. Satellite imagery was obtained from Google Earth Engine using the Harmonized Sentinel-2 MSI Level-2A Surface Reflectance dataset (COPERNICUS/S2_SR_HARMONIZED), and processed within ArcGIS Pro (version 3.6.0, Esri; <https://www.esri.com/>).

Table S1. The training and validation performance of the 15 candidate models.

|  | ***Loss Function*** | ***Validation IoU*** | ***Validation OA (%)*** | ***Validation Loss*** | ***Training IoU*** | ***Training OA (%)*** | ***Training Loss*** |
| --- | --- | --- | --- | --- | --- | --- | --- |
| ***ResUNet34*** | BCE + Dice | 0.85 | 0.92 | 0.66 | 0.79 | 0.90 | 0.76 |
|  | BCE | 0.88 | 0.94 | 0.19 | 0.78 | 0.90 | 0.25 |
|  | Dice | 0.75 | 0.86 | 1.34 | 0.59 | 0.75 | 1.47 |
|  | Focal + Dice | 0.88 | 0.93 | 1.45 | 0.72 | 0.85 | 1.60 |
|  | Focal | 0.71 | 0.83 | 0.03 | 0.73 | 0.88 | 0.03 |
| ***DeepLabV3+*** | BCE + dice | 0.78 | 0.88 | 0.68 | 0.75 | 0.88 | 0.79 |
|  | BCE | 0.93 | 0.96 | 0.15 | 0.75 | 0.88 | 0.28 |
|  | Dice | 0.83 | 0.91 | 1.36 | 0.61 | 0.77 | 1.48 |
|  | Focal + Dice | 0.56 | 0.72 | 1.58 | 0.78 | 0.90 | 1.57 |
|  | Focal | 0.79 | 0.88 | 0.02 | 0.64 | 0.84 | 0.03 |
| ***ResNet18*** | BCE + Dice | 0.82 | 0.90 | 0.77 | 0.79 | 0.90 | 0.78 |
|  | BCE | 0.74 | 0.85 | 0.36 | 0.79 | 0.90 | 0.27 |
|  | Dice | 0.77 | 0.87 | 1.35 | 0.62 | 0.78 | 1.51 |
|  | Focal + Dice | 0.80 | 0.89 | 1.55 | 0.76 | 0.88 | 1.58 |
|  | Focal | 0.55 | 0.72 | 0.04 | 0.72 | 0.88 | 0.03 |

Table S2. The test set performance of the five top-performance candidate models at each of the three test locations.

|  | ***Model*** | ***Loss Function*** | ***Overall Accuracy (%)*** | ***Producer Accuracy (%)*** | ***User Accuracy (%)*** | ***IoU*** |
| --- | --- | --- | --- | --- | --- | --- |
| ***Big Bend*** | DeepLabV3+ | Dice | 0.89 | 0.76 | 0.98 | 0.75 |
|  | DeepLabV3+ | BCE | 0.59 | 0.05 | 0.96 | 0.05 |
|  | ResUNet | Focal + Dice | 0.89 | 0.74 | 1.00 | 0.74 |
|  | ResUNet | BCE | 0.82 | 0.59 | 1.00 | 0.59 |
|  | ResUNet | BCE + Dice | 0.68 | 0.27 | 1.00 | 0.27 |
| ***Jamaica Bay*** | DeepLabV3+ | Dice | 0.97 | 0.83 | 0.78 | 0.67 |
|  | DeepLabV3+ | BCE | 0.96 | 0.59 | 0.85 | 0.53 |
|  | ResUNet | Focal + Dice | 0.98 | 0.85 | 0.86 | 0.75 |
|  | ResUNet | BCE + Dice | 0.97 | 0.74 | 0.87 | 0.66 |
|  | ResUNet | BCE | 0.97 | 0.66 | 0.94 | 0.63 |
| ***Point aux Chenes*** | DeepLabV3+ | BCE | 0.92 | 0.70 | 0.91 | 0.66 |
|  | DeepLabV3+ | Dice | 0.92 | 0.82 | 0.81 | 0.69 |
|  | ResUNet | BCE + Dice | 0.96 | 0.86 | 0.92 | 0.80 |
|  | ResUNet | BCE | 0.95 | 0.85 | 0.92 | 0.80 |
|  | ResUNet | Focal + Dice | 0.95 | 0.79 | 0.94 | 0.75 |
